# Phase I study of onapristone, a type I antiprogestin, in female patients with previously treated recurrent or metastatic progesterone receptor-expressing cancers

**DOI:** 10.1371/journal.pone.0204973

**Published:** 2018-10-10

**Authors:** Paul H. Cottu, Jacques Bonneterre, Andrea Varga, Mario Campone, Alexandra Leary, Anne Floquet, Dominique Berton-Rigaud, Marie-Paule Sablin, Anne Lesoin, Keyvan Rezai, François M. Lokiec, Catherine Lhomme, Jacques Bosq, Alice S. Bexon, Erard M. Gilles, Stefan Proniuk, Veronique Dieras, David M. Jackson, Alexander Zukiwski, Antoine Italiano

**Affiliations:** 1 Department of Medical Oncology, Institut Curie, Paris, France; 2 Department of Medical Oncology, Centre Oscar Lambret, Lille, France; 3 Department of Medical Oncology, Gustave Roussy, Villejuif, France; 4 Department of Medical Oncology, Institut de Cancérologie de l'Ouest—René Gauducheau, Nantes, France; 5 Department of Medical Oncology, Institut Bergonié, Bordeaux, France; 6 Department of Medical Oncology, Centre Rene Huguenin-Institut Curie, St Cloud, France; 7 Bexon Clinical Consulting, Upper Montclair, NJ, United States of America; 8 Invivis Pharmaceuticals, Bridgewater, NJ, United States of America; 9 Arno Therapeutics, Flemington, NJ, United States of America; University of Manchester, UNITED KINGDOM

## Abstract

**Introduction:**

Onapristone is a type I progesterone receptor (PR) antagonist, which prevents PR- mediated DNA transcription. Onapristone is active in multiple preclinical models and two prior studies demonstrated promising activity in patients with breast cancer. We conducted a study of extended release (ER) Onapristone to determine a recommended dose and explore the role of transcriptionally-activated PR (APR), detected as an aggregated subnuclear distribution pattern, as a predictive biomarker.

**Methods:**

An open-label, multicenter, randomized, parallel-group, phase 1 study (target n = 60; NCT02052128) included female patients ≥18 years with PR^pos^ tumors. APR analysis was performed on archival tumor tissue. Patients were randomized to five cohorts of extended release (ER) onapristone tablets 10, 20, 30, 40 or 50 mg BID, or immediate release 100 mg QD until progressive disease or intolerability. Primary endpoint was to identify the recommended phase 2 dose. Secondary endpoints included safety, clinical benefit and pharmacokinetics.

**Results:**

The phase 1 dose escalation component of the study is complete (n = 52). Tumor diagnosis included: endometrial carcinoma 12; breast cancer 20; ovarian cancer 13; other 7. Median age was 64 (36–84). No dose limiting toxicity was observed with reported liver function test elevation related only to liver metastases. The RP2D was 50 mg ER BID. Median therapy duration was 8 weeks (range 2–44), and 9 patients had clinical benefit ≥24 weeks, including 2 patients with APR^pos^ endometrial carcinoma.

**Conclusion:**

Clinical benefit with excellent tolerance was seen in heavily pretreated patients with endometrial, ovarian and breast cancer. The data support the development of Onapristone in endometrial endometrioid cancer. Onapristone should also be evaluated in ovarian and breast cancers along with APR immunohistochemistry validation.

## Trial registration

clinicaltrials.gov, NCT02052128

## Introduction

Expression of the progesterone receptor (PR) has been described in breast [1,2], endometrial [3,4], prostate [5,6], ovarian [7], and several other cancers [8–10]. Antiprogestins have been shown to have an inhibitory effect on the growth of different type of cancer cells, and antiprogestin treatment has been studied in breast [11], endometrial [12] and prostate cancers [13], and in uterine sarcomas [14].

The effects of progesterone are mediated by two distinct nuclear receptor proteins, PRA and PRB, which are two transcriptional isoforms of the single PR gene. In luminal epithelial cells of the normal breast and in normal endometrium, both PR isoforms are expressed and are required to mediate the physiological effects of progestin ligands [15,16]. The two PR isoforms have both been detected in malignant tissues, such as breast, endometrial, ovarian and prostate cancers [17].

Onapristone (ONA) is a type I antiprogestin which prevents the PRA and PRB monomers from dimerizing, inhibits ligand-induced phosphorylation and prevents association of the PR with its co-activators, thus preventing PR-mediated DNA transcription [18]. In contrast to other antiprogestins, ONA does not allow the PR complex to bind to DNA, minimally modulates PR-mediated genes, and inhibits ligand-induced PR phosphorylation [19,20]. Activity has been shown in several preclinical models, including endometrial cancer [21] and the clinical anticancer activity of ONA has been previously documented in patients with hormone therapy-naïve [22] or tamoxifen-resistant [23] breast cancer (BC). Transcriptionally activated PR (APR) can be detected by evaluation of the subnuclear distribution pattern using immunohistochemistry (IHC). Using this method, APR is being explored as a predictive IHC biomarker in endometrioid cancer of the uterus, and is under development as a potential companion diagnostic to identify patients more likely to respond to ONA [24].

Early clinical studies employing an immediate release (IR) formulation of ONA have shown that ONA is well-tolerated except for abnormalities in liver functional tests (LFT) [22,23,25–27]. Using highly-purified drug substance, an extended-release (ER) oral formulation of ONA was developed aiming at achieving continuous exposure and constant PR suppression. The high purity and the expected reduced maximum plasma concentration (C_max)_ may mitigate the impact on LFT elevations seen with the previous IR formulation.

The current study was designed to assess the safety and tolerability of ONA ER tablets with pharmacokinetics data, as well as to determine the recommended dose to be used in future clinical studies.

## Materials and methods

### Eligibility

Inclusion criteria included post-menopausal female patients ≥ 18 years, previously treated for recurrent or metastatic PR-expressing cancer (e.g. endometrial (EC), ovarian (OC), breast cancers (BC) or uterine sarcoma), with evaluable disease per RECIST 1.1, available tissue blocks or biopsy specimens to determine PR and APR status, Eastern Cooperative Oncology Group (ECOG) performance status 0–1, and signed informed consent. PR determination for study inclusion was performed locally on archived tissue blocks. Central PR/APR evaluation was performed retrospectively.

Other key exclusion criteria included: creatinine clearance < 60 mL/min, total bilirubin > upper limit of normal (ULN), alkaline phosphatase > ULN (or > 2.5 x ULN with liver or > 5 x ULN with bone metastases), ALT/AST > ULN (or > 2.5 x ULN with liver metastases), QTcF > 480 msec, chronic inflammatory liver condition, severe concomitant disease, uncontrolled brain metastases, inadequate washout from previous therapy, inability to swallow or absorb tablets, use of inhibitors, inducers or substrates of CYP3A4, or use of progestin-based hormone replacement therapy.

### Study design and treatment

**Fig 1:**
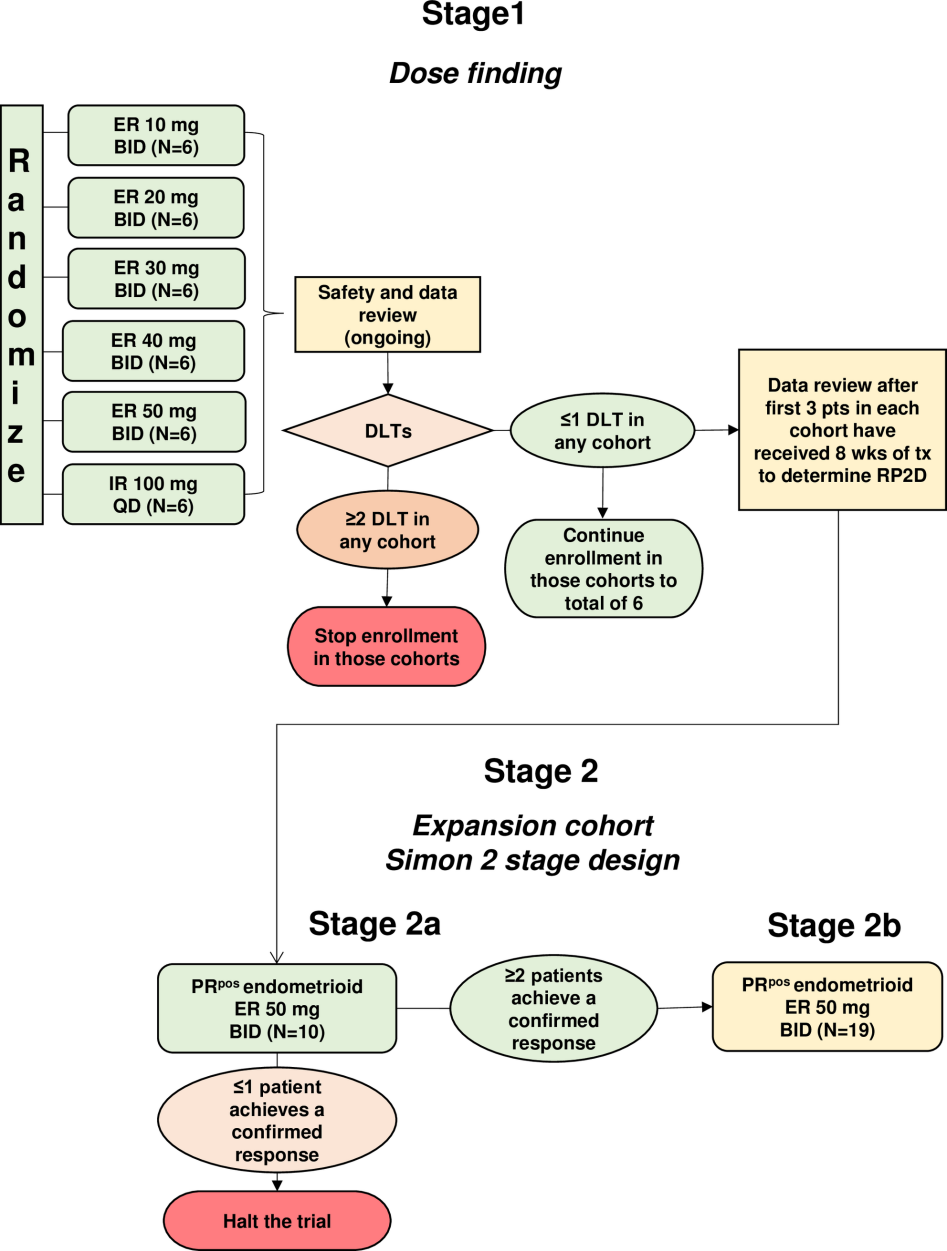
Study design. Flow chart of the two parts of the study.

This was an open-label, multicenter, randomized, parallel-group, phase 1–2 study; the phase I part is reported here. The trial was conducted in five centers in France (NCT 02052128). To determine the recommended phase II dose (RP2D), patients enrolled in this phase I study were randomized to six cohorts: five cohorts of ER ONA tablets (10 mg BID, 20 mg BID, 30 mg BID, 40 mg BID, 50 mg BID) and one cohort using the IR tablet formulation (100 mg QD). The randomized design was used in view of previous experience with ONA doses up to 400 mg/day [26], and considering that a total dose of 100 mg/day would not be exceeded, patients would not be in jeopardy of taking a potentially toxic ER dose. The study was planned to include approximately 60 female patients with PR^pos^ tumors, including a 20 patient expansion cohort at the RP2D dose (Fig 1).

The trial has been approved by the Ile de France III Comité pour la Protection des Personnes (a French National Ethics Committee), the ANSM (*Agence Nationale de Sécurité du Médicament*, French regulatory authority), and individual sites Institutional Review Boards. A written informed consent was obtained from each study patient.

ONA ER tablets were produced utilizing highly purified drug substance with release kinetics of 10–12 hours depending on tablet dose. An 8-week dose-limiting toxicity (DLT) observation period was utilized to characterize thoroughly the safety profile, as previous ONA studies demonstrated a spike in LFTs at approximately 6 weeks of treatment. The protocol was later amended to include a formal phase 2 study in patients with recurrent or metastatic APR^pos^ endometrioid uterine cancer treated at the RP2D and the sample size increased by 30 additional patients. This ongoing phase 2 part will be later reported (NCT02052128).

Patients were treated until documented progressive disease (PD) or intolerance to medication. The study design was in agreement with guidance for phase 1 dose escalation protocols [28].

### Study endpoints

The primary endpoints were to determine the RP2D of single agent ONA ER, and DLTs during an 8-week observation period.

Secondary endpoints were to: compare the safety profiles of ONA ER and IR formulations, study the pharmacokinetics (PK) of the ER and IR formulations, and evaluate potential anticancer efficacy based on tumor response and progression-free survival (PFS).

Exploratory endpoints included determination of the relationship between PR, APR and efficacy.

### Safety and tolerability

A data review committee (DRC) was set, involving five independent members (two oncologists, one pharmacovigilance specialist (chair), one statistician, one clinical pharmacologist), as well as the lead principal investigator. The DRC charter outlined responsibilities including declaration of the RP2D. The sponsor provided the DRC with tables, listings and data summary for each meeting.

Adverse events (AEs), including abnormal laboratory test results, were collected until 30 days after the last ONA dose. LFTs were monitored weekly for the first 8 weeks, then every 2 weeks. DLT was defined as a confirmed AE of Common Terminology Criteria for Adverse Events (CTCAE) Version 4 grade (G) ≥3 within the first 8 weeks of treatment with a reasonable chance to be related to ONA, by determination of the DRC.

### Efficacy

Tumor assessments were performed every 8 weeks and evaluated according to RECIST v.1.1.

### Pharmacokinetics

Blood samples were collected at 0, 1, 2, 3, 4, 6, 8, 12 (before next BID dose), and 24 (before next dose- for 100 mg IR only) hours post ONA dosing, as well as at hour 0 on days 8, 29 and 57 (just before drug intake). Plasma concentrations of ONA, mono-demethylated ONA (M1) and other metabolites in plasma and urine were analyzed with a validated ultra-performance liquid chromatography with tandem mass spectrometry detection (UPLC-MS/MS) assay. Pharmacokinetic modeling was performed using Monolix software V4.1 in order to estimate the following PK parameters: C_max_ (maximum plasma concentration), T_max_ (time to maximum plasma concentration), AUC_0-last_, AUC_0-8_ (AUC: area under curve), t_1/2_ (Half-life), V_d_ (volume of distribution), CL (clearance), and V_c_ (Volume of central compartment) [29].

### Biomarkers

IHC detection of PRA and PRB was centrally performed on 3–4 μm sections of archival tumor tissues, using a sequential staining procedure. APR status was determined using commercially available isotype-specific antibodies to PRA and PRB. Methods for APR determination have been published separately [24]. Briefly, commercial antibodies anti PRA (Novocastra 16, Leica Biosystems, Newcastle-upon-Tyne, UK) and anti PRB (Novocastra SA N27, Leica Biosystems, Newcastle-upon-Tyne, UK) were used according to the manufacturer specifications. If a sample contained at least 10% PRA positive or PRB positive cells, the tumor was considered to be PRA^pos^ or PRB ^pos^. The global PR status was also evaluated with the 1A6 antibody (Abcam, Paris, France). APR^pos^ was defined as any tumor with >5% countable tumor cells with the aggregated pattern.

## Results

This manuscript reports the results of the completed dose escalation phase 1 study.

### Patients

**Fig 2:**
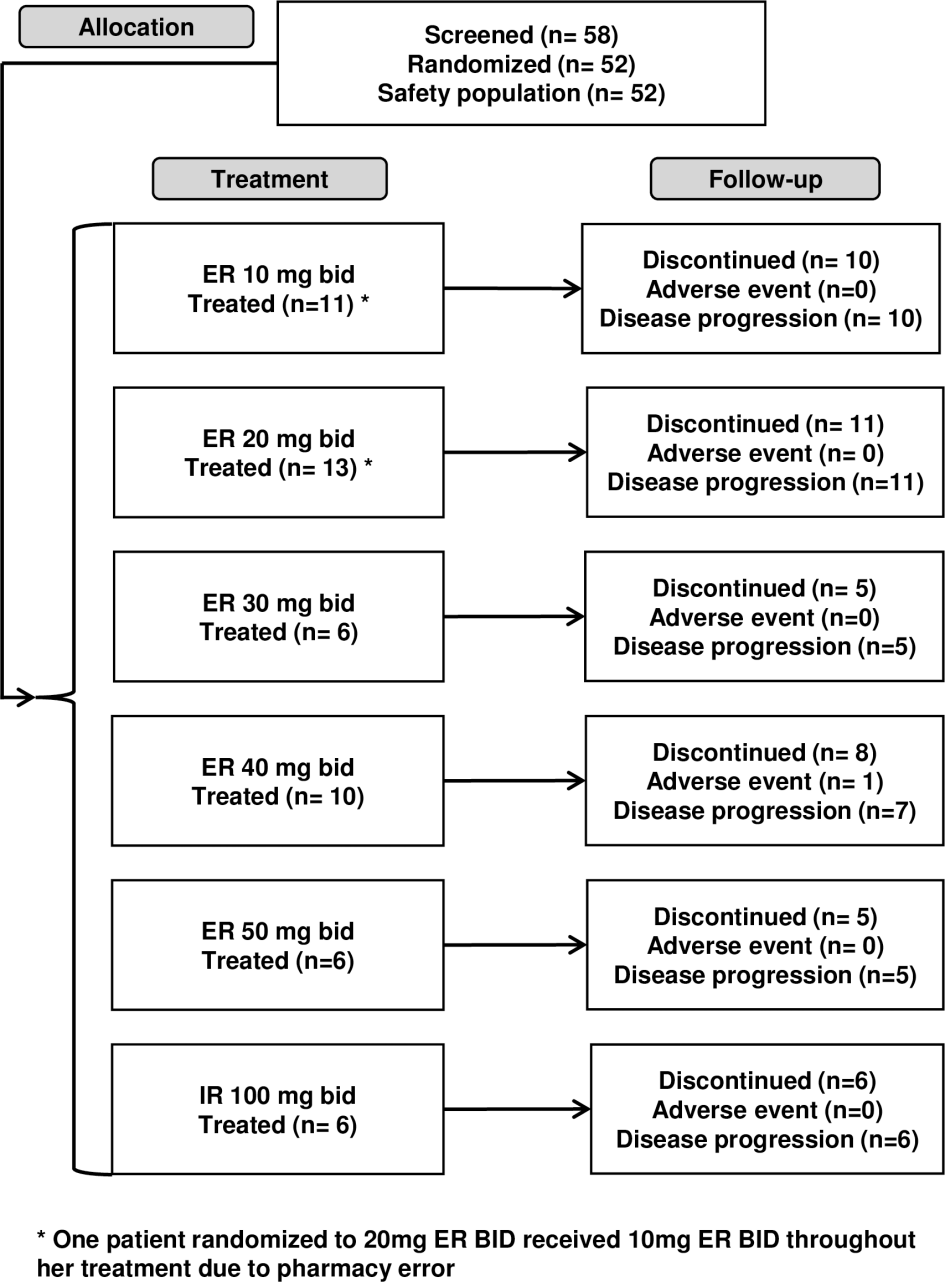
CONSORT diagram.

Of 58 patients screened, 52 were randomized and treated over a 10-month period in 2014. (Fig 2)

Patients and disease characteristics are depicted in Table 1. All patients were of Caucasian descent. The median age was 64 years, median weight was 66 kg, and all patients had ECOG status of 0 or 1.

**Table 1:** Demographic data and disease characteristics.

		Onapristone Dose					
	Overall (n = 52)	10 mg BID (n = 12 ^a^)	20 mg BID (n = 12 ^a^)	30 mg BID (n = 6)	40 mg BID (n = 10)	50 mg BID (n = 6)	100 mg QD (n = 6)
Age (years)							
Median	64	67	63	65	65	63	61
Range	36–84	42–81	36–78	59–68	45–82	46–84	53–77
Race n(%)							
White	52 (100)	12 (100)	12 (100)	6 (100)	10 (100)	6 (100)	6 (100)
BMI (kg/m^2^)							
Median	25 ^b^	25	23	26 ^b^	25	25	26
Range	18–35 ^b^	19–35	18–35	20–32 ^b^	20–35	20–26	22–29
Weight (kg)							
Median	66	68	63	65	68	61	65
Range	40–91	45–86	40–78	50–91	46–90	52–66	51–72
ECOG status [n(%)]							
0	23 (44)	7 (58)	4 (33)	2 (33)	3 (30)	4 (67)	3 (50)
1	29 (56)	5 (42)	8 (67)	4 (67)	7 (70)	2 (33)	3 (50)
Primary tumor [n(%)]							
Breast	20 (38)	5 (42)	5 (42)	2 (33)	5 (50)	3 (50)	0
Ovarian	13 (25)	4 (33)	3 (25)	1 (17)	4 (40)	1 (17)	0
Endometrium	13 (25)	3 (25)	3 (25)	1 (17)	0	1 (17)	5 (83)
Other ^c^	6 (12)	0	1 (8)	2 (33)	1 (10)	1 (17)	1 (17)
Metastatic sites [n (%)]							
Lymph nodes	28 (54)	7 (58)	6 (50)	3 (50)	5 (50)	3 (50)	4 (67)
Liver	26 (50)	6 (50)	6 (50)	4 (67)	6 (60)	3 (50)	1 (17)
Bone	23 (44)	5 (42)	5 (42)	5 (83)	4 (40)	4 (67)	0
Peritoneum	20 (39)	4 (33)	7 (58)	2 (33)	4 (40)	1 (17)	2 (33)
Lung	15 (29)	3 (25)	5 (42)	2 (33)	1 (10)	2 (33)	2 (33)
Pleura	8 (15)	3 (25)	2 (17)	0	2 (20)	0	1 (17)
Ovarian	2 (4)	1 (8)	1 (8)	0	0	0	0
Spleen	4 (8)	1 (8)	0	1 (17)	0	0	2 (33)
Other	16 (31)	4 (33)	4 (33)	1 (17)	4 (40)	1 (17)	(33)

a. 1 patient randomized to 20mg was treated at 10mg BID and is included in all data tables as being at the 10mg dose level

b. 1 patient missing BMI (minor protocol deviation)

c. 2 uterine leiomyosarcomas, 2 endometrial stromal sarcomas, 1 uterine cancer, 1 vertex apocrine carcinoma

The most common tumors (n) were: BC (20), OC (13), and EC (13) (Table 1). The most common sites of metastases were lymph nodes (54%) and liver (50%). Dose cohorts ER-30mg BID and ER-40mg BID had a slight over-representation of patients with liver metastases, whereas dose cohort IR 100 mg QD had only one patient with liver metastases (17%). All patients were heavily pre-treated; prior treatments included [mean (range)]: chemotherapy [4 (1–11)], endocrine therapy [1 (1–7)], biologic/small molecule therapy [1 (1–2)], and radiotherapy [1 (1–3)].

### Safety and tolerability

A minimum of 6 patients were treated at each dose level for at least 8 weeks. The median duration of ONA treatment was 12.7 weeks (range 4–53).

No DLT was observed. Only transient elevations in LFTs occurred, mostly in patients with liver metastases and abnormal LFTs at baseline. Fifty-one patients discontinued ONA treatment for disease progression, and one for an AE (blood bilirubin G3 elevation, eventually deemed progression of disease in the liver). Seven patients (13%) experienced transient dose interruptions for AEs (1 each: nausea, ALT increase, gastroenteritis, GGT increased, thoracic pain, post procedural cellulitis, LDH increase, abdominal pain and atrial fibrillation).

Fifty-one patients (98%) experienced at least one treatment emergent adverse event (TEAE). The most common (>10%) TEAEs regardless of relationship to treatment and grade appear in Table 2. Those TEAEs considered related to ONA were reported in 33 (64%) patients. The most common (>10%) drug related TEAEs of any grade were asthenia (25%), increased GGT (19%), increased ALT and AST (14% each),and nausea (12%). There was no obvious relationship between ONA dose and observed AEs.

**Table 2:** All TEAEs >10%–safety population.

		Onapristone Dose					
Preferred Term	Overall (N = 52) n (%)	10 mg BID (N = 12 ^a^) n (%)	20 mg BID (N = 12 ^a^) n (%)	30 mg BID (N = 6) n (%)	40 mg BID (N = 10) n (%)	50 mg BID (N = 6) n (%)	100 mg QD (N = 6) n (%)
**Any TEAE**	**51 (98)**	**12 (100)**	**12 (100)**	**6 (100)**	**9 (90)**	**6 (100)**	**6 (100)**
Asthenia	27 (52)	6 (50)	6 (50)	4 (67)	5 (50)	3 (50)	3 (50)
GGT increase	18 (35)	5 (42)	3 (25)	2 (33)	5 (50)	2 (33)	1 (17)
AST increase	11 (21)	5 (42)	2 (17)	1 (17)	2 (20)	1 (17)	0
ALT increase	10 (19)	3 (25)	2 (17)	1 (17)	2 (20)	0	2 (33)
Nausea	9 (17)	3 (25)	1 (8)	2 (33)	1 (10)	1 (17)	1 (17)
ALP increase	8 (15)	3 (25)	1 (8)	2 (33)	2 (20)	0	0
Constipation	9 (17)	0	6 (50)	0	1 (10)	1 (17)	1 (17)
Abdominal pain	9 (17)	2 (17)	3 (25)	1 (17)	0	1 (17)	2 (33)
Vomiting	6 (12)	0	2 (17)	1 (17)	1 (10)	1 (17)	1 (17)
Pyrexia	9 (17)	0	3 (25)	2 (33)	2 (20)	0	2 (33)
Diarrhea	6 (12)	2 (17)	2 (17)	1 (17)	0	1 (17)	0
Peripheral edema	6 (12)	2 (17)	1 (8)	2 (33)	1 (10)	0	0
Hyperkalemia	6 (12)	2 (17)	1 (8)	0	1 (10)	1 (17)	1 (17)
Cough	6 (12)	0	1 (8)	2 (33)	0	1 (17)	2 (33)
Arthralgia	5 (10)	2 (17)	0	0	0	1 (17)	(33)

a. 1 patient randomized to 20mg was treated at 10mg BID and is included in all data tables as being at the 10mg dose level

Thirty (58%) patients experienced at least one TEAE ≥ G3 (Table 3). In ten patients (19%), TEAEs considered related to ONA included: increased GGT (13%), increased AST and ALP (6% each), increased bilirubin (4%), and increased ALT and LFTs, asthenia and pulmonary embolism (2% each). All except two of these TEAEs were associated with progressive disease: one G3 GGT elevation at week 12 in a responding patient, lasting 1 month with associated transient G1 AST and bilirubin increase, with no clinical symptoms; and the other a G3 GGT elevation at week 3, lasting 3 weeks, in a patient with liver metastases and baseline G1 GGT elevation. Both elevations decreased spontaneously with no action taken. None of the G3 AST, ALT or bilirubin elevations were considered to be dose limiting by the DRC due to the presence of concurrent progressive disease in the liver.

**Table 3:** Grade ≥ 3 (CTCAE) TEAEs in > 1 patient.

		Onapristone Dose					
Preferred Term	Overall (N = 52) n (%)	10 mg BID (N = 12 ^a^) n (%)	20 mg BID (N = 12 ^a^) n (%)	30 mg BID (N = 6) n (%)	40 mg BID (N = 10) n (%)	50 mg BID (N = 6) n (%)	100 mg QD (N = 6) n (%)
**Any Grade ≥ 3 TEAE**	**30 (58)**	**8 (67)**	**7 (58)**	**3 (50)**	**6 (60)**	**3 (50)**	**3 (50)**
GGT increase	13 (25)	5 (42)	2 (17)	2 (33)	3 (30)	1 (17)	0
Asthenia	4 (8)	0	2 (17)	0	1 (10)	0	1 (17)
ALP increase	4 (8)	1 (8)	1 (8)	0	2 (20)	0	0
AST increase	3 (6)	2 (17)	1 (8)	0	0	0	0
ALT increase	2 (4)	1 (8)	1 (8)	0	0	0	0
Bilirubin increase	2 (4)	1 (8)	0	0	1 (10)	0	0
Disease progression	2 (4)	0	1 (8)	0	1 (10)	0	0
Peripheral edema	2 (4)	1(8)	0	0	1 (10)	0	0
Pyrexia	2 (4)	0	2 (17)	0	0	0	0

a. 1 patient randomized to 20mg was treated at 10mg BID and is included in all data tables as being at the 10mg dose level

The only treatment-related serious AEs were G3 LFT elevations (n = 4; 8%), all associated with disease progression in the liver as reviewed by the DRC. These occurred across dose cohorts: 10 mg BID (AST increased, bilirubin increased), 20 mg BID (LFTs abnormal), and 40 mg BID (bilirubin increased). No relationship was found between AEs and study drug exposure.

No treatment-related deaths were reported. One patient died within 30 days of last dose (respiratory distress syndrome due to progressive lung metastases). No other significant AEs attributable to the mechanism of action were recorded.

### Pharmacokinetics

ONA AUC and C_max_ were dose-proportional across dose levels, including the 100 mg IR formulation, with high correlation coefficients (R^2^ values are 0.76 and 0.97, respectively, see S1 and S2 Figs). Average T_max_ was 3.01 hours (2.71–3.2) vs 1.84 hours for ER vs IR formulations, respectively, and concentrations of drug were sustained longer with a 60% (+/- 20) relative bioavailability for ER vs IR formulation. Steady state for the ER formulation was attained before day 8, and the mean ONA minimum concentrations at steady state were up to 5 times those obtained at day 1; day 8 through levels were similar to day 1 for IR. There was no evidence of ONA accumulation at day 57. The observed mean t_1/2_ for the ER formulation was approximately 18.01 hours (range, 13.9 to 37), consistent with steady state achievement before day 8. ONA plasma concentration versus time curves suggest biphasic elimination (S3 Fig). Variability for onapristone PK is moderate and greater for the IR versus the ER formulation. The average V_d_ value was 5.41 L (standard error: 25), while the average Vc value was 41.1 L (standard error: 45).

There was no correlation between AUC and safety events for the ER vs IR formulations. Higher exposure was associated with a better disease control (see below). Consequently, the recommended dose to take into future trials was declared 50 mg BID of the ER formulation, which was determined by the DRC to be safe and well tolerated.

### Biomarkers

Fifty-two (100%) patient tumors were PR^pos^ by local laboratory testing. When evaluated centrally by IHC with the bispecific 1A6 antibody, 12 (23%) were PR^neg^, 41 (79%) were PR^pos^, and 1 (2%) was of unknown PR status due to missing samples. With PR isoforms specific antibodies, PRA ^pos^ or PRB ^pos^ tumors were identified in respectively 81%, 75% and 92% of EC, BC and OC patients, and in 60% of other cancer patients by central testing. APR^pos^ tumors were identified in 62% of ECs, 30% of BCs, 15% of OCs, and 0% in the other cancers (S4 Fig and S2 Table).

### Efficacy

Progressive disease was most frequently observed (62–83%) in the 10 mg and 20 mg BID ER and 100 mg QD IR dose cohorts, and less frequently observed (40–50%) in the 30 mg, 40 mg and 50 mg BID ER cohorts; conversely, stable disease (SD) + partial response rates were highest (50–60%) in the 30 mg, 40 mg and 50 mg BID ER dose cohorts, and lowest (17–31%) in the 10 mg and 20 mg BID ER and 100 mg QD IR cohorts. The overall median progression free survival (PFS) was 58 days (range 57–92) and clinical benefit rate (CBR) was achieved in 17% of patients. (Table 4).

**Table 4:** Tumor response.

Response by Cohort		Onapristone Dose					
Response (RECIST) n (%)	Overall (N = 52)	10 mg BID (N = 11)	20 mg BID (N = 13)	30 mg BID (N = 6)	40 mg BID (N = 10)	50 mg BID (N = 6)	100 mg QD (N = 6)
CBR (PR + SD ≥ 24 weeks)	9 (17)	1 (9)	2 (16)	2 (33)	2 (20)	2 (33)	0
Objective Response	1 (2)*	1 (9)*	0	0	0	0	0
CR	0	0	0	0	0	0	0
PR	1 (2)	1 (9)*	0	0	0	0	0
SD	20 (38)	2 (18)	5 (38)	3 (50)	6 (60)	3 (50)	1 (17)
SD ≥ 24 weeks	8 (15)	0	2 (16)	2 (33)	2 (20)	2 (33)	0
PD	31 (60)	8 (73)	8 (62)	3 (50)	4 (40)	3 (50)	5 (83)
PFS in days median (95% CI)	58 (57–92)	57 (22–112)	54 (24–197)	85 (57–342)	113 (24–169)	77 (54–301)	58 (43–113)

* Duration of response 225 days (7.5 months)

Clinical benefit was observed in ovarian, breast and uterine endometrioid cancers (Table 5).

**Table 5:** Tumor response by tumor type.

Response by tumor type n (%)	Breast N = 20	Ovarian N = 13	Uterine endometrioid N = 13	Sarcoma / other N = 6
CBR (PR + SD ≥ 24 weeks)	3 (15)	4 (33)	2 (15)	0
PR	0	1 (8)	0	0
SD	7 (35)	7 (58)	4 (31)	2 (33)
SD ≥ 24 weeks	3 (15)	3 (25)	2 (15)	0
PD	13 (65)	5 (38)	9 (69)	4 (67)

One patient with serous OC experienced a PR for 32 weeks and 8 patients had SD for at least 24 weeks (detailed in Table 6). Most interestingly, both patients with endometrioid EC experiencing clinical benefit had APR^pos^ tumors, as well as the 3 patients with APR^pos^ breast cancer (Tables 6 and S2).

**Table 6:** Patients with objective response and clinical benefit ≥ 24 weeks.

Tumor type	PR % retest	APR status	#Prior Rx	Dur Prev Rx, months	Metastases	Dose	Response	% change STL	Dur weeks
Serous OC	0	Neg	3	8	LN	10	Part Resp	-52	32
Serous OC	0	Neg	5	6	LN	50	SD	-7	28
Granulosa OC	A: 20 B: 20	Neg	3	5	LN, Liver, peritoneum	40	SD	+17.6	24
Granulosa OC	A: 80 B: 70	Neg	4	Unk	Liver, perito- neal colon	30	SD	+31	32
EC	A: 80 B: 60	Pos	4	3	Lung, bone	30	SD	+26	49
EC	A: 60 B: 70	Pos	3	7	Pelvis, lung	20	SD	+22	32
BC	A: 50 B: 50	Pos	7	11	Liver, bone	50	SD	-	43
BC	A: 90 B: 90	Pos	3	-	Bone	20	SD	-	28
BC	A: 60 B: 40	Pos	7	4	Liver, bone	40	SD	+80	24

A: local testing; B: central testing;, Rx–treatments, Dur = duration, Prev = previous, STL = sum of target lesions SD = stable disease, Part Resp = Partial Response; PR = Progesterone Receptor, APR = Activated Progesterone Receptor

## Discussion

Antiprogestins have been used in reproductive medicine and more recently recognized as having anti-tumor activity in gynecological cancers [30]. Historical data suggest that antiprogestin therapy has potential to be useful in advanced BC [11,22,31]. Preclinical data also suggest antiproliferative activity in OC and EC cell lines [32]. Specifically, mifepristone has been clinically evaluated in breast and ovarian carcinomas, showing clear signs of activity [33]. Lonaprisan was tested in a phase 2 study as second-line therapy in metastatic BC but did not meet the planned objective response rate [11], which may have been due to lack of stratification according to patients molecular profiles [31].

ONA is a type I antiprogestin developed by a previous sponsor for benign gynecological indications. Three studies in BC were conducted, two of which indicated potential efficacy, the third was not reported. A phase 2 study in tamoxifen-resistant BC patients (n = 101) showed CBR of 49% with median duration of CR+PR 11 months and median duration of SD 7 months [23]. A phase 2 study in hormone-therapy-naïve BC patients (n = 19) resulted in CBR of 67% and median duration of objective response and SD 70 weeks [22]. A phase 3 study comparing ONA to megestrol acetate was unfortunately discontinued after accruing approximately 100 patients, due to termination of the development program [23].

In the previous ONA development program, LFT elevations were considered a concern in the planned indications, triggering the need to develop alternative formulas. In our study, the most common perturbation of liver function was GGT elevation, which was the only change seen in the absence of progressive liver disease and was not included in the DLT definition due to its lack of clinical impact. Reformulation of highly purified drug substance as an ER formulation appears to have succeeded in reducing both the incidence and the degree of LFT elevations (AST, ALT and bilirubin). All the cases of clinically meaningful LFT elevation observed in this study were associated with progressive hepatic metastases. The ER BID formulation may have decreased the potential for liver toxicity thanks to a more constant exposure and with a lower C_max_.

In the present study, ONA was exceptionally well tolerated, with no DLT reported. The PK of ONA was biphasic and dose proportional. No relationship between AEs and exposure was detected with the ER formulation.

Tumor assessments strongly suggested anticancer efficacy, even in heavily pretreated patients. Nine of the 52 patients had a clinical benefit lasting at least 24 weeks, and 11 additional patients experienced stable disease as best response. Although PK was dose-proportional, there was no hint of relationship between dose level and toxicity. Based on these data, the highest ER dose regimen (50 mg BID) was declared by the DRC to become the RP2D.

We still lack clear predictive markers of efficacy with antiprogestin agents. It has been suggested that the degree of antagonistic activity is dependent on the balance among co-activators and co-repressors regulating the transcriptional activity of the PR, as well as the ratio of PRA/PRB isoforms [30]. The interaction with other key steroid receptors such as the Estrogen receptor is still matter of debate [34]. A new potential predictive biomarker assay for APR [24] has been developed and the correlation between APR status and efficacy was evaluated on archived specimens in a mixed patient population; no strong correlation was found between efficacy and a positive APR assessment. The APR biomarker test has been analytically validated only in endometrial endometrioid cancer, where it performed well. It continues to require refinement and validation in other tumor types such as breast and ovarian cancer, as our present results strongly suggest its potential validity is these settings. However, the APR test is still to be considered in development and cutoffs and thus positivity may change. Our preliminary interpretation might change with validation of the test in different tumor types and with more data.

Of note, although 100% of patients were PR^pos^ on local laboratory testing, only 79% were positive on central review. Possible reasons for this include: many of the tissue specimens sent for central review were different from those used in the original local pathology review (50% primary tumor, 50% metastases), heterogeneity in the tumors across different disease sites, and known issue of PR staining reproducibility [35,36].

## Conclusion

The new ER formulation of ONA was well tolerated and resulted in meaningful clinical benefit in heavily pretreated patients with ovarian, breast and uterine endometrioid cancers. There were no grade 3–4 LFT elevations in the absence of progressive liver metastases, and no new safety signals were observed. Pharmacokinetics data, showing that the ER formulation is dose proportional and causes less variability than the IR formulation, support use of the ER administration to mitigate LFT elevations. Data supports development of ER ONA at 50 mg BID in APR^pos^ uterine endometrioid cancer, with clinical validation of the APR diagnostic. ONA should also be evaluated in ovarian and breast cancers along with APR IHC validation.

## Supporting information

S1 Fig Dose proportionality: AUC. Mean AUC values (area under curve) are plotted against the initial dose. AUC is highly correlated to the initial dose (r^2^ = 0.76)
(PPTX)

S2 Fig Dose proportionality: C_max_. For each evaluated patient (black dots), the individual C_max_ (maximum plasma concentration after the first dose of onapristone) is plotted against the initial dose. C_max_ is highly correlated to the initial dose (r^2^ = 0.97)
(PPTX)

S3 Fig Pharmacokinetics modeling curves. Pharmacokinetics modeling curves for the 100 mg dose level are shown. (A) 50 mg ER BID. (B) 100 mg IR QD. X axis: hours after first dose. Y axis: plasma concentration (ng/mL).
(PPTX)

S4 Fig PR expression patterns. Patterns of progesterone receptor expression in endometrial carcinoma cells. (A) Activated (aggregated) pattern of PR expression (red arrows). (B) Diffuse pattern of PR expression (black arrow).
(PPTX)

S1 Table Pharmacokinetics raw data. Sheet 1 contains the individual PK data, for each dose level. These data are summarized in sheet 2.
(XLSX)

S2 Table PR expression data. Sheet 1 contains PR status raw data. Sheet 2 contains the summary of PR and APR status.
(XLSX)

S1 File Protocol amendments. (DOCX)

S2 File CONSORT Checklist. (DOCX)

S3 File Protocol. (PDF)

## Abbreviations

ALP: Alkaline phosphatase
ALT: alanine aminotransferase
ANSM: Agence Nationale de Sécurité du Médicament
APR: Activated progesterone receptor
AST: aspartate aminotransferase
AUC: Area under curve
BC: Breast cancer
BID: *bis in die*, twice a day
BL: baseline
BMI: Body mass index
CBR: Clinical benefit rate
C_max_: maximum plasma concentration
CR: Complete response
DLT: Dose limiting toxicity
DRC: Data Review Committee
EC: Endometrial carcinoma
ER: Extended release
GGT: Gamma-glutamyl transferase
IHC: Immunohistochemistry
IR: Immediate release
LFT: Liver functional tests
NE: Not evaluable
OC: Ovarian cancer
ONA: Onapristone
PD: Progressive disease
PK: Pharmacokinetics
PR: Partial response
PR: Progesterone receptor
QD: *quaque die*, once a day
QTcF: QT interval with Fridericia correction
RP2D: Recommended phase II dose
SD: Stable disease
STL: Sum of target lesions
T_1/2_: Half life
TEAE: treatment emergent adverse event
T_max_: time to maximum plasma concentration
ULN: Upper limit of normal
V_c_: Volume of central compartment
V_d_: Volume of distribution
