# Supplementary figures and images for: Phase I study of onapristone, a type I antiprogestin, in female patients with previously treated recurrent or metastatic progesterone receptor-expressing cancers
Source: PLoS One. 2018 Oct 10;13(10):e0204973. doi: 10.1371/journal.pone.0204973 (PMC6179222; doi:10.1371/journal.pone.0204973)

## Slide 1
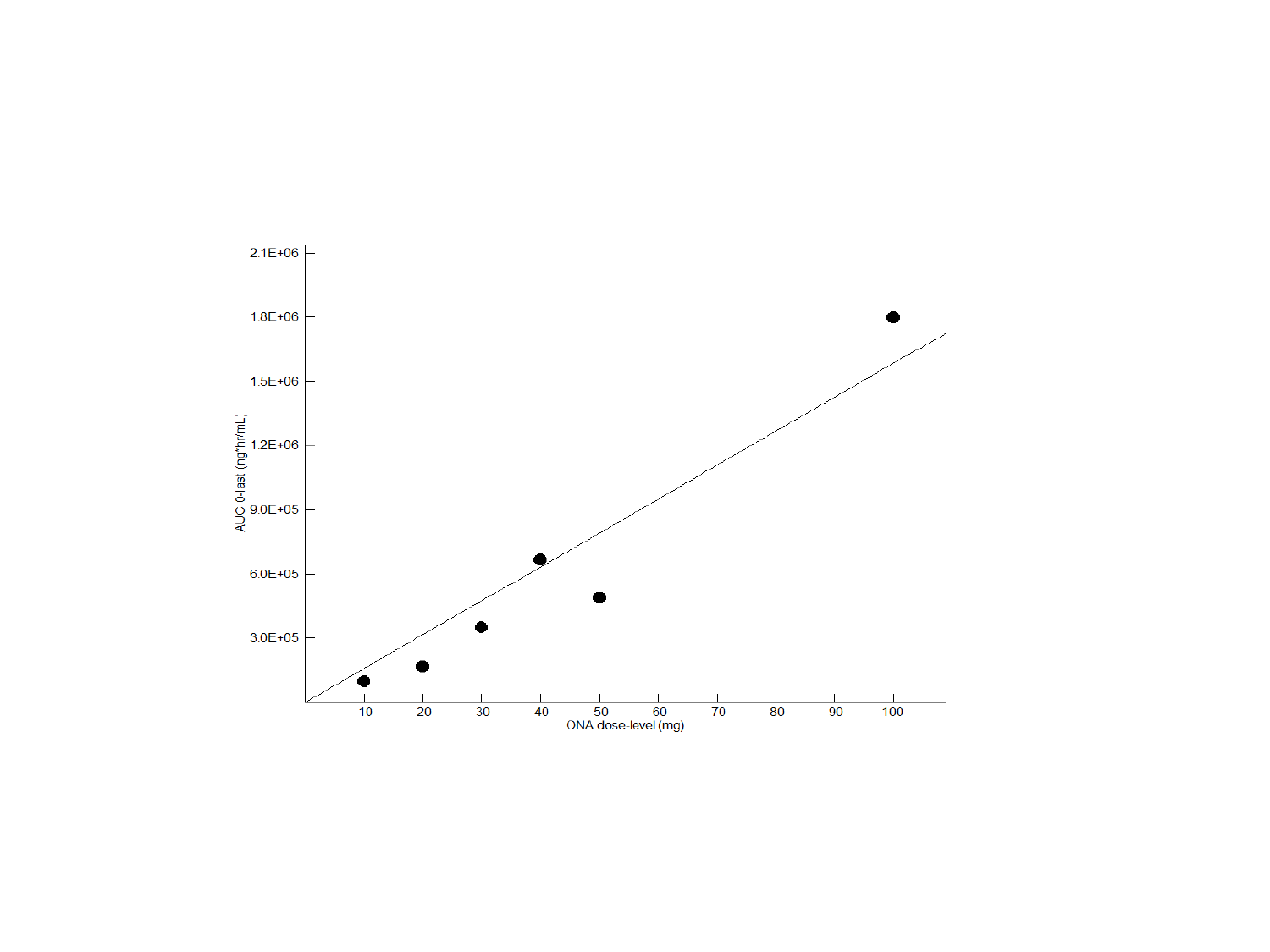

Supplement: S1 Fig — Mean AUC values (area under curve) are plotted against the initial dose. AUC is highly correlated to the initial dose (r2 = 0.76) (PPTX) [file pone.0204973.s001.pptx]

## Slide 1
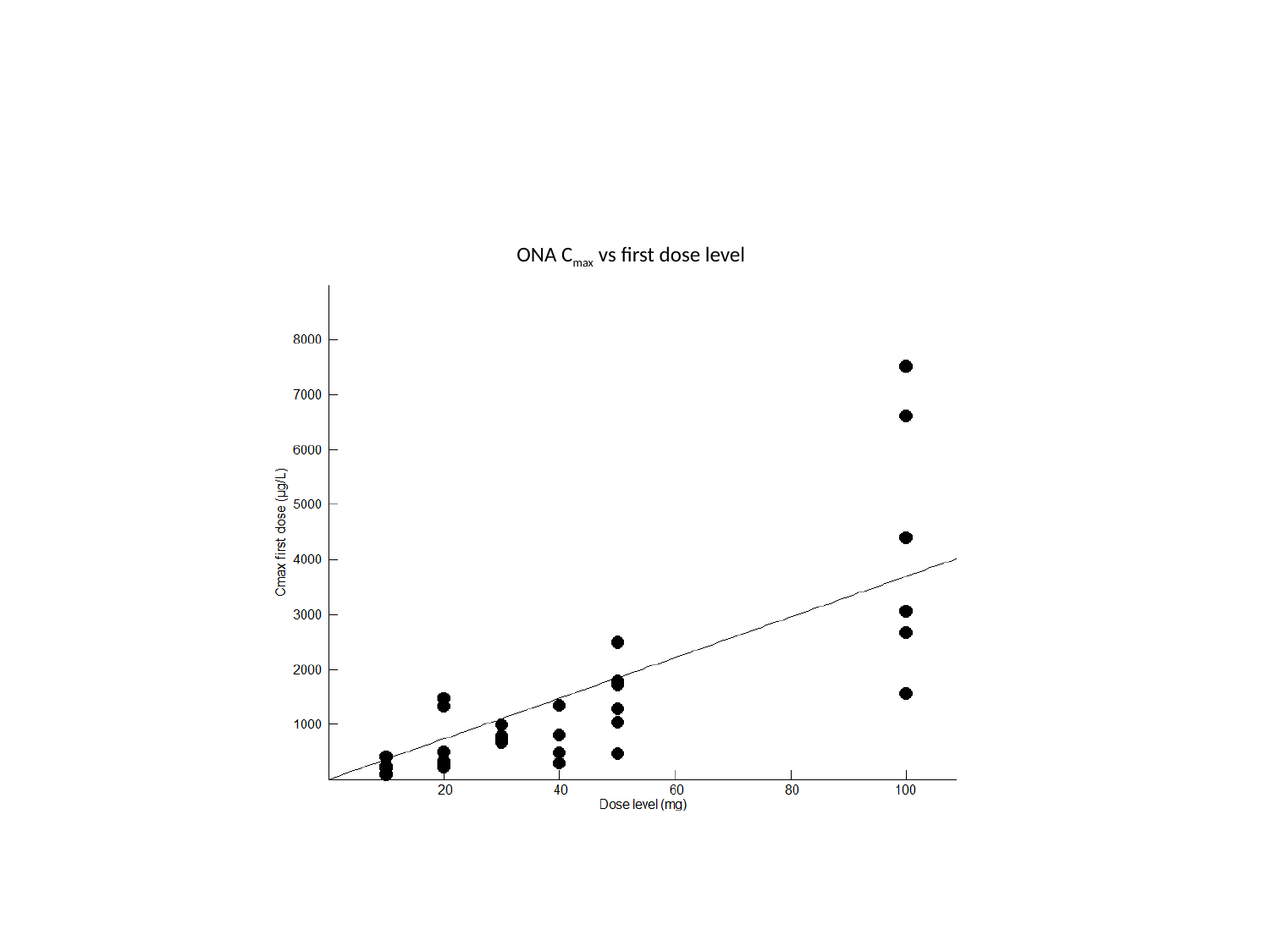

ONA Cmax vs first dose level

Supplement: S2 Fig — For each evaluated patient (black dots), the individual Cmax (maximum plasma concentration after the first dose of onapristone) is plotted against the initial dose. Cmax is highly correlated to the initial dose (r2 = 0.97) (PPTX) [file pone.0204973.s002.pptx]

## Slide 1
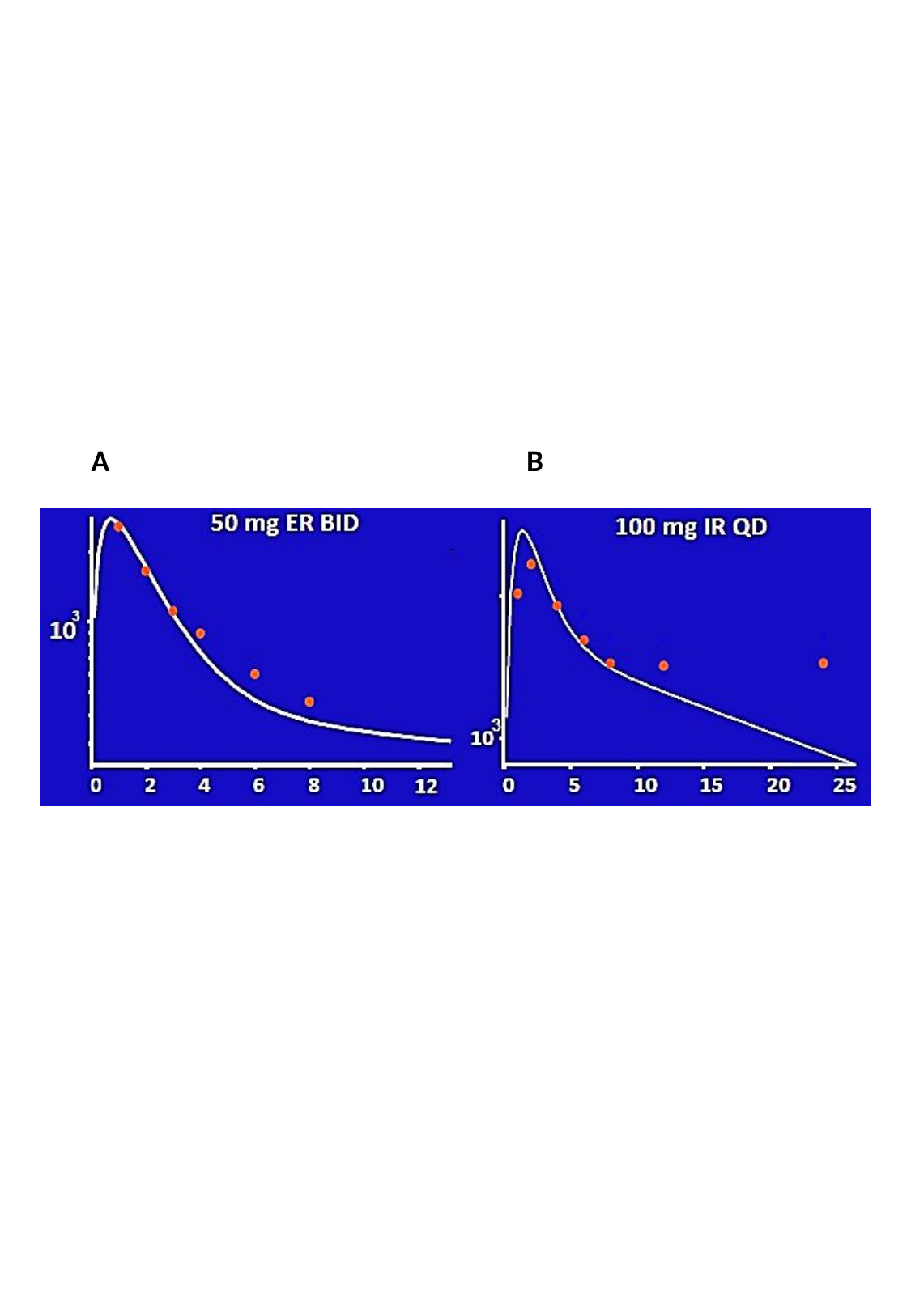

A
B

Supplement: S3 Fig — Pharmacokinetics modeling curves for the 100 mg dose level are shown. (A) 50 mg ER BID. (B) 100 mg IR QD. X axis: hours after first dose. Y axis: plasma concentration (ng/mL). (PPTX) [file pone.0204973.s003.pptx]

## Slide 1
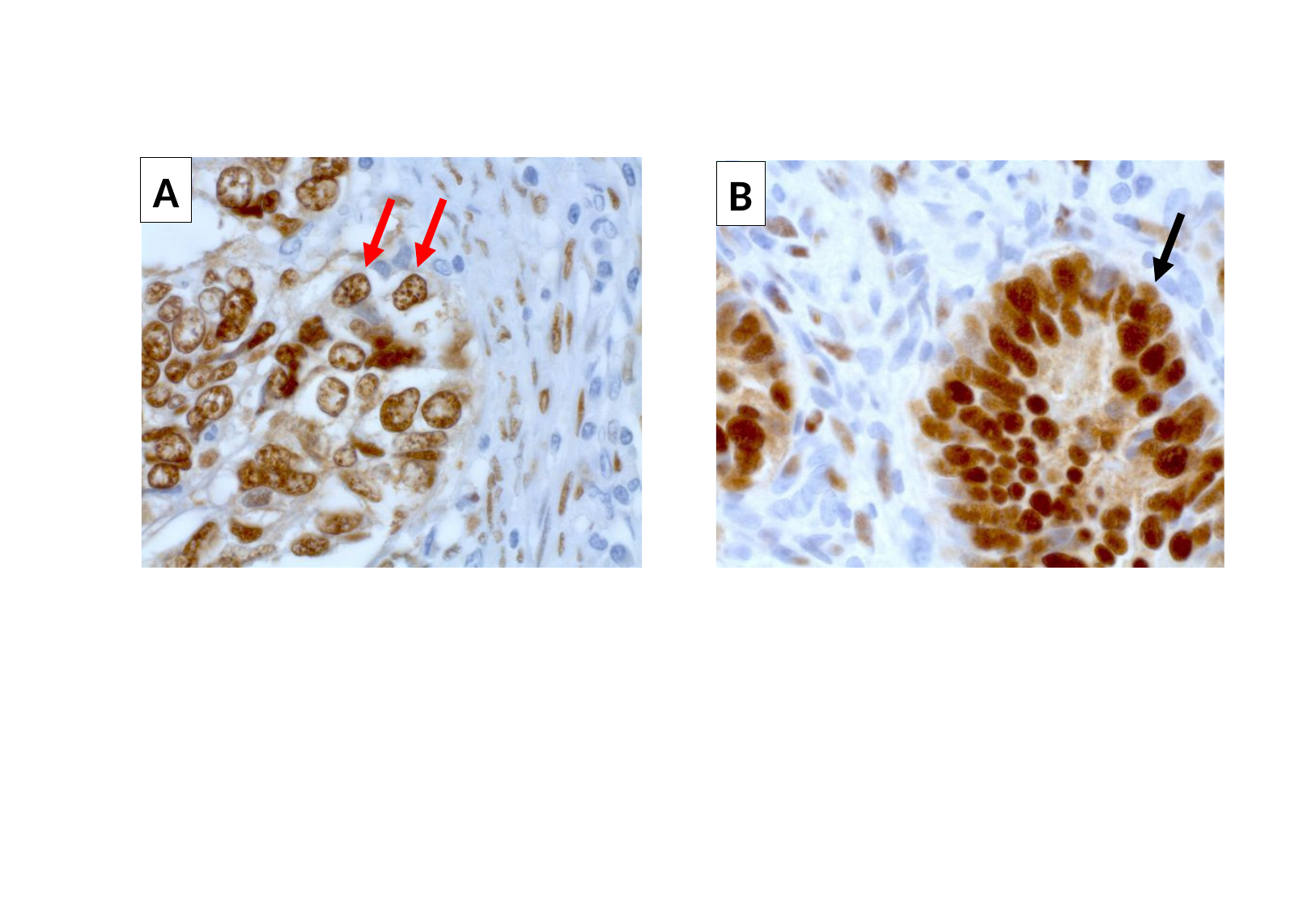

A
B

Supplement: S4 Fig — Patterns of progesterone receptor expression in endometrial carcinoma cells. (A) Activated (aggregated) pattern of PR expression (red arrows). (B) Diffuse pattern of PR expression (black arrow). (PPTX) [file pone.0204973.s004.pptx]
