# Supplementary material for: Phase I study of onapristone, a type I antiprogestin, in female patients with previously treated recurrent or metastatic progesterone receptor-expressing cancers
Source: PLoS One. 2018 Oct 10;13(10):e0204973. doi: 10.1371/journal.pone.0204973 (PMC6179222; doi:10.1371/journal.pone.0204973)
Supplement: S1 File — (DOCX) [file pone.0204973.s007.docx]

| **Reason for Change:** New Investigators added | | |
| --- | --- | --- |
| **Section** | **New text** | **Old text** |
| **LIST OF CONTACTS** | **Dr Andrea Leary**  Institut Gustave Roussy  Consultation De Gynécologie Médicale  114 Rue Edouard Vaillant  VILLEJUIF CEDEX 94805  France  Phone: +33 1 42114377  Fax: +33 1 42115214  Email: [Alexandra.leary@gustaveroussy.fr](mailto:Alexandra.leary@gustaveroussy.fr)  **Pr Mario Campone**  1-Institut de Cancérologie de l’OUEST  Bd Jacques Monod  44805 Saint Herblain-Nantes cedex  Tel:+33240679977  Fax:+33240679776  2-Centre de Recherche du Cancer Nantes-Angers: UMR-INSERM U892/CNRS 6299  3-Faculté de Médecine d’Angers  E-mail: [mario.campone@ico.unicancer.fr](mailto:mario.campone@ico.unicancer.fr)  **Dr Thierry Lesimple**  Head, Clinical Research Unit  Head, Brittany Melanoma Network  Medical Oncology Department  Comprehensive Cancer Center Eugène Marquis  CS 44229  35042 Rennes Cedex  France  Phone: +33 2 99 25 31 95  Fax: +33 2 99 25 32 33  Email: [t.lesimple@rennes.unicancer.fr](mailto:t.lesimple@rennes.unicancer.fr)  **Dr Antoine Italiano**  Institut Bergonie  Early Phase Trials and Sarcoma Units  229 cours de l'Argonne  33000 Bordeaux  Phone: + 33 5 56 33 33 33  Fax: + 33 5 56 33 33 53  Mobile: + 33 6 09 42 08 13  E-mail: [a.italiano@bordeaux.unicancer.fr](mailto:a.italiano@bordeaux.unicancer.fr) | (not applicable) |
| **Reason for Change:** Add 3 hour PK blood sample to be consistent with other sections of protocol | | |
| **Section** | **New text** | **Old text** |
| Synopsis - Criteria for Evaluation, PK | Plasma concentrations of onapristone, and mono-demethylated onapristone (M1) on day 1 at H0, 1, 2, **3,** 4, 6, 8, 12 (before next dose) and 24 (before next dose), and days 8, 29 and 57 at H0. | Plasma concentrations of onapristone, and mono-demethylated onapristone (M1) on day 1 at H0, 1, 2, 4, 6, 8, 12 (before next dose) and 24 (before next dose), and days 8, 29 and 57 at H0. |
| 9.2 Baseline: Day 1 | - Obtain blood samples for PK on day 1 at H0, 1, 2, **3,** 4, 6, 8, 12 (before next BID dose). | - Obtain blood samples for PK on day 1 at H0, 1, 2, 4, 6, 8, 12 (before next BID dose). |
| **Reason for Change:** Clarifying the time period for definition of DLT | | |
| **Section** | **New text** | **Old text** |
| Synopsis - Study Design, Stage 1 | - If there are 2 or more DLTs in a cohort, that cohort will stop enrolling. DLT is defined as a confirmed grade ≥3 AE per CTCAE **within the first 8 weeks of treatment with a reasonable chance to be related to the study drug based upon the determination of the DRC**. If there is ≤1 DLT in any cohort, that cohort will continue enrolling up to 6 pts. If any of the LFT tests is elevated to grade ≥3, the medical monitor will immediately assess the complete LFT profile and other safety data, discuss it with the investigator and the event will be classified as an isolated laboratory abnormality or a liver DLT AE. For example, an isolated grade 3 AST elevation together with a grade 2 ALT elevation may not be considered a DLT, but requires repeat examination and continued monitoring per protocol. All liver function abnormalities will be reviewed by the data review committee. If DLTs are not observed at the highest dose level of ER onapristone and efficacy is not observed up to that dose level, the protocol may be amended to further escalate the dose. | - If there are 2 or more DLTs in a cohort, that cohort will stop enrolling. DLT is defined as a confirmed grade ≥3 AE per CTCAE. If there is ≤1 DLT in any cohort, that cohort will continue enrolling up to 6 pts. If any of the LFT tests is elevated to grade ≥3, the medical monitor will immediately assess the complete LFT profile and other safety data, discuss it with the investigator and the event will be classified as an isolated laboratory abnormality or a liver DLT AE. For example, an isolated grade 3 AST elevation together with a grade 2 ALT elevation may not be considered a DLT, but requires repeat examination and continued monitoring per protocol. All liver function abnormalities will be reviewed by the data review committee. If DLTs are not observed at the highest dose level of ER onapristone and efficacy is not observed up to that dose level, the protocol may be amended to further escalate the dose. |
| **Section** | **New text** | **Old text** |
| 6.1. Description of Overall Study Design and Plan, Stage 1 | DLT is defined as confirmed grade ≥3 AE **per CTCAE within the first 8 weeks of treatment with a reasonable chance to be related to the study drug based upon the determination of the DRC**. If there is ≤1 DLT in any cohort, that cohort will continue enrolling up to 6 patients. If any of the LFT tests is elevated to grade ≥3, the medical monitor will immediately assess the complete LFT profile and other safety data, discuss it with the investigator and the event will be classified as an isolated laboratory abnormality or a liver DLT AE. For example, an isolated grade 3 AST elevation together with a grade 2 ALT elevation may not be considered a DLT, but requires repeat examination and continued monitoring per protocol. All liver function abnormalities will be reviewed by the data review committee. | DLT is defined as confirmed grade ≥3 AE. If there is ≤1 DLT in any cohort, that cohort will continue enrolling up to 6 patients. If any of the LFT tests is elevated to grade ≥3, the medical monitor will immediately assess the complete LFT profile and other safety data, discuss it with the investigator and the event will be classified as an isolated laboratory abnormality or a liver DLT AE. For example, an isolated grade 3 AST elevation together with a grade 2 ALT elevation may not be considered a DLT, but requires repeat examination and continued monitoring per protocol. All liver function abnormalities will be reviewed by the data review committee. |
| **Reason for Change:** Clarifying time for disease follow-up | | |
| **Section** | **New text** | **Old text** |
| Appendix 1 Schedule of Assessments – Footnote 9 | **^9^ If the patient discontinues treatment for reasons other than PD, radiological follow-up will be performed every 12 weeks until disease progression. If the patient discontinues for PD, no further efficacy evaluations are required.** | ^9^ ~~Non PD patients only~~. |
