# Supplementary material for: Phase I study of onapristone, a type I antiprogestin, in female patients with previously treated recurrent or metastatic progesterone receptor-expressing cancers
Source: PLoS One. 2018 Oct 10;13(10):e0204973. doi: 10.1371/journal.pone.0204973 (PMC6179222; doi:10.1371/journal.pone.0204973)
Supplement: S3 File — (PDF) [file pone.0204973.s009.pdf]

CLINICAL STUDY PROTOCOL

**Protocol Title:**	**A Phase I Study of Onapristone in Patients with Progesterone Receptor Expressing Cancers**
**EudraCT no.**	2013-002859-14
**Protocol Number:**	ARN-AR18-CT-101
**Development Phase:**	1
**Study Sponsor:**	Arno Therapeutics, Inc<br>200 Route 31 North Suite 104<br>Flemington, NJ 08822<br>USA
**Protocol Version:**	1.0
**Version Date:**	28 August 2013

ARNO THERAPEUTICS APPROVAL

Alex Zukiwski, MD
Chief Medical Officer
Arno Therapeutics, Inc.

28-Aug-2013
Date

INVESTIGATOR PROTOCOL AGREEMENT PAGE

I agree:

- To assume responsibility for the proper conduct of the study at this site.
- To conduct the study in compliance with this protocol, any future amendments, and with any other study conduct procedures provided by Arno Therapeutics Inc.
- Not to implement any changes to the protocol without written agreement from Arno Therapeutics Inc., and prior review and written approval from the Comite de Protection des Personnes (CPP [Ethics Committee]) except where necessary to eliminate an immediate hazard to study patients.
- That I am thoroughly familiar with the appropriate use of the study drug (onapristone), as described in this protocol and any other information provided by Arno Therapeutics Inc., including, but not limited to, the current Investigator's Brochure (IB).
- That I am aware of, and will comply with, good clinical practices (GCP) and all applicable regulatory requirements.
- To ensure that all persons assisting me with the study are adequately informed about the Arno Therapeutics Inc., study drug and of their study-related duties and functions as described in the protocol.

Signature: ____ Date: ____________________

Name (print): ____

Site Number:

LIST OF CONTACTS

**Sponsor and Medical Monitor Contact:**	**Alex Zukiwski, MD**<br>Chief Medical Officer<br>Arno Therapeutics, Inc<br>200 Route 31 North Suite 104<br>Flemington, NJ 08822<br>USA<br>Tel: 001-862-703-7170<br>az@arnothera.com
**Study Manager/Clinical Research Associate:**	**Andre Paquin, CTM**<br>**Samuel Vorin, CRA**<br>Biotrial Rennes<br>7-9 rue Jean-Louis Bertrand<br>35000 Rennes<br>France<br>Tel [+33] (0)2 99 599 191<br>Andre.Paquin@biotrial.com
**Contract Research Organization:**	**Biotrial Rennes**<br>7-9 rue Jean-Louis Bertrand<br>35000 Rennes<br>France<br>Tel [+33] (0)2 99 599 191
**Clinical Investigators:**	**Dr Paul H. Cottu**<br>Institut Curie<br>Oncologie Medicale<br>26 Rue d'Ulm<br>Paris 75005<br>France<br>Phone: [+33] (0)14 4324000 ext 4670<br>Fax: [+33] (0)153104041<br>Email: paul.cottu@curie.net

Dr Andrea Varga

Department of Medicine,

Service des Innovations Therapeutiques Precoces (SITEP),

Institut Gustave Roussy,

Villejuif, Universite Paris XI,

France

Phone: [+33] (0)1 42 11 42 96

Fax: [+33] (0)1 42 11 53 00

Email : andrea.varga@igr.fr

Clinical Pharmacology:

Fran□ois Lokiec, PharmD, PhD

Olivier Madar, PharmD

Keyvan Rezai, PhD

Radiopharmacology Department

Institut Curie-H6pital Rene-Huguenin

35, Rue Dailly

92210 St Cloud, France

+33(0)1 47 11 16 15

francois.lokiec@curie.net

keyvan.rezai@curie.net

olivier.madar@curie.net

I. SYNOPSIS

**Title of Study:**	**A Phase I Study of Onapristone in Patients with Progesterone Receptor Expressing Cancers**
**Protocol Number:**	ARN-AR-18CT-101
**EudraCT Number:**	2013!002859!14
**Clinical Investigational Unit:**	Multicenter
**Phase of Development:**	1
**Objectives:**	• Determine the recommended phase 2 dose (RP2D) of a single agent extended-release tablet formulation of oral onapristone for future clinical development.<br>• Determine the safety profile of extended-release oral onapristone tablets administered on a chronic BID schedule.<br>• Determine the safety profile of an oral 100 mg immediate-release tablet formulation of onapristone on a chronic QD schedule.<br>• Compare the safety of the extended-release tablets BID and immediate-release tablets QD.<br>• Study the PK of chronic daily dosing of the extended-release and immediate-release onapristone tablet formulations.<br>• Evaluate potential anti-cancer efficacy at each dose level.<br>• Exploratory enumeration and testing of progesterone receptors (PR) in circulating tumor cells (CTCs) to inform companion diagnostic development.<br>• Exploratory collection of plasma for exosome analysis.
**Study Design:**	• Multi-center, open-label, randomized, parallel group two-stage phase 1 study with an expansion component.<br>• Stage 1: Six dose cohorts using the extended-release tablet formulation (10 mg BID, 20 mg BID, 30 mg BID, 40 mg BID, 50 mg BID) and the immediate-release tablet formulation (100 mg QD) will be randomized in parallel. Six patients are planned to be enrolled in each cohort. Data review will be

undertaken in an ongoing manner by the medical monitor. The data review committee will review the safety, PK and PD data after the first patient at each dose level has completed 4 weeks of therapy, after the 2nd patient at each dose level has completed 4 weeks of therapy and after the 3rd patient at each dose level has completed 4 weeks of therapy. In addition the data review committee will be convened to review any dose-limiting toxicities (DLTs).

- If there are 2 or more DLTs in a cohort, that cohort will stop enrolling. DLT is defined as a confirmed grade □3 AE per CTCAE. If there is :S1 DLT in any cohort, that cohort will continue enrolling up to 6 pts. If any of the LFT tests is elevated to grade □3, the medical monitor will immediately assess the complete LFT profile and other safety data, discuss it with the investigator and the event will be classified as an isolated laboratory abnormality or a liver DLT AE. For example, an isolated grade 3 AST elevation together with a grade 2 ALT elevation may not be considered a DLT, but requires repeat examination and continued monitoring per protocol. All liver function abnormalities will be reviewed by the data review committee. If DLTs are not observed at the highest dose level of ER onapristone and efficacy is not observed up to that dose level, the protocol may be amended to further escalate the dose.
- Stage 2: The data review committee will be convened when 3 patients have been treated for 8 weeks at each dose level. This data review will be conducted to assess safety, efficacy and PK, with the intent of recommending a dose level to enroll additional patients. Once the RP2D has been determined, that dose cohort will be expanded by enrollment of an additional 20 patients, to a total of 26 patients to confirm the safety profile. If additional data beyond the first 3 patients at each dose level is required, further data committee meetings will be convened until a RP2D can be recommended.
- LFTs will be monitored weekly for the first 8 weeks then every 2 weeks.
- Plasma PK samples will be taken on days 1, 8, 29 and 57 in at least 3 patients at each dose level and urine PK on day 1. Additional PK (plasma and urine) samples will be collected in the event of LFT elevation.
- Blood samples will be drawn in all patients at baseline and every 4-8 weeks until PD, to enumerate and determine PR

	status in CTCs.<br>• Tumor assessments will be performed every 8 weeks according to RECIST 1.1.<br>• Safety assessment of each dose level will use a cutoff at day 57 (8 weeks of treatment) for determination of the RP2D.<br>• Two dose reductions (to 75% and 50% of original dose) will be permitted for patients where continuation of therapy is thought to be in the patient's best interest, with the lowest permitted onapristone dose being 5 mg BID.<br>• Tumor blocks or fresh tumor tissue will be collected from all patients to allow verification of PR status/type and APR status (see Appendix 6).
**Selection of Patients:**	Inclusion Criteria:<br>Patients meeting all of the following inclusion criteria at screening/day-1 of treatment will be eligible for participation in the study.<br>1. Post-menopausal female patients, 18 years of age or greater (see definition of post-menopausal in Section 7.2).<br>2. Recurrent or metastatic PR-expressing cancer which has the potential to benefit from an anti-progestin treatment including but not limited to endometrial cancer, ovarian, or breast cancer or uterine sarcoma.<br>3. Patients who have metastatic or recurrent disease after previous surgery, radiation therapy, and/or chemotherapy are eligible. No restriction is placed on the number of prior therapies.<br>4. Evaluable disease per RECIST 1.1.<br>5. Appropriate archival AND current tissue blocks or biopsy specimens to determine ER/PR and APR status (see Appendix 6). An appropriate tissue sample is one which gives a reasonable chance of reflecting the PR status of the tumor.<br>6. Signed, written informed consent must be obtained and documented according to ICH-GCP, the local regulatory requirements, and local data protection laws prior to study-specific screening procedures.<br>7. ECOG performance status 0-1.<br>8. Health care coverage.

Exclusion Criteria:

Patients meeting any of the following exclusion criteria at Screening/day -1 of treatment will not be enrolled in the study.

1. Calculated creatinine clearance of <60 mL/min (based on the Cockcroft-Gault equation, see Appendix 8).
2. Patients with any other prior malignancy are not allowed except for the following:
3. Adequately treated basal cell or squamous cell skin cancer
4. In situ cervical cancer
5. Adequately treated Stage I or II cancer from which the patient is currently in complete remission or other cancer from which the patient has been disease-free for 2 years
6. Body mass index (BMI) <18.5 or >35 kg/m^2.
7. On ECG a QTc(F) interval >480 msec or any clinically significant cardiac rhythm abnormalities.
8. Liver function tests documented within the screening period and on day -1 of treatment period:
   - Total bilirubin > ULN (except in patients diagnosed with Gilbert's disease).
   -Alkaline phosphatase > UNL or > 2.5 x UNL in case of liver metastases, or > 5 x UNL in case of bone metastases.
   - ALT/AST > UNL or > 2.5 x UNL in case of liver metastases.
9. Known positive virology/serology for human immunodeficiency virus (HIV)-1, HIV-2, hepatitis B (surface antigen), or hepatitis C (testing not required).
10. Chronic inflammatory liver condition. History or clinical evidence of any liver or biliary pathology including cirrhosis, infectious disease, inflammatory conditions, steatosis, or cholangitis (including ascending cholangitis, primary sclerosing cholangitis, obstruction, perforation, fistula of biliary tract, spasm of sphincter of Oddi, biliary cyst or biliary atresia).
11. Chronic adrenal failure or is receiving concurrent long-term corticosteroid therapy.
12. History or clinical evidence of any surgical or medical condition which the investigator judges as likely to interfere with the results of the study or pose an additional risk in participating e.g., active or clinically significant history of disease involving a major organ system—vascular, cardiac,

	uncontrolled hypertension, pulmonary, gastrointestinal, gynecologic, hematologic, neurologic, neoplastic, renal, endocrine or immunodeficiency, or clinically significant active psychiatric disorders.<br>13. Used any prescription medication during the prior 1 month that the investigator judges is likely to interfere with the study or to pose an additional risk to the patient in participating, specifically inhibitors or inducers of cytochrome P450 (CYP)3A4 (see Appendix 4).<br>14. Received an investigational product or been treated with an investigational device within 30 days prior to first drug administration, or plans to start any other investigational product or device study within 30 days after last drug administration.<br>15. Received prior systemic anticancer treatment (chemotherapy, targeted therapies including kinase inhibitors, antibodies, etc) less than 5 half-lives before the first dose of study drug or radiotherapy within 30 days; toxicity of the anticancer treatment must have recovered to grade 1 or less.<br>16. Current progestin-based hormone replacement therapy. If a patient is on progestins, including hormone replacement therapy and natural products known to contain progestins, they must be stopped 7 days prior to beginning study treatment.<br>17. Lack of physical integrity of the upper gastrointestinal tract, malabsorption syndrome, or inability to swallow pills.<br>18. Has a mental incapacity or language barriers precluding adequate understanding, co-operation, and compliance with the study requirements.<br>19. Is, in the judgment of the investigator, unable or unwilling to comply with the requirements of the study.<br>20. Uncontrolled brain metastases or treatment by neurosurgical resection or brain biopsy within 4 weeks prior to Day 1.
**Planned Sample Size:**	Approximately 60 patients
**Investigational Therapy:**	Onapristone extended-release tablets 10 mg BID, 20 mg BID, 30 mg BID, 40 mg BID or 50 mg BID; onapristone immediate-release tablets 100 mg QD
**Reference Therapy:**	N/A

**Treatment Duration:**	Until intolerable safety issue not addressable by dose interruption and reduction or treatment failure, or if patient elects to withdraw, or for non-compliance with either protocol-specified evaluations or onapristone treatment
**Primary Endpoint:**	• Determination of the RP2D of oral extended-release onapristone tablets utilizing a day 57 safety cut off.
**Secondary Endpoints:**	• Safety and tolerability of extended-release onapristone tablets BID and of immediate-release onapristone tablets QD.<br>• Comparison of safety of extended-release BID vs. immediate-release QD schedules.<br>• Efficacy based on tumor assessments (RECIST 1.1) and dates of progression.<br>• PK of onapristone, mono-demethylated onapristone and other metabolites in plasma and urine.
**Exploratory Endpoints:**	• Enumeration and analysis of progesterone receptor and activated PR in circulating tumor cells.<br>• Exosome analysis for gene signature determination.
**Criteria for Evaluation:**	Safety:<br>AEs and SAEs (including abnormal laboratory test results) will be collected from the time of signing the informed consent until 30 days after the last onapristone dose. Only AEs/abnormalities occurring after the first dose of onapristone will be considered treatment-emergent.<br>Weekly LFT laboratory samples should preferably be drawn at the study sites rather than at an external laboratory.<br>Efficacy:<br>Tumor assessments per RECIST 1.1 (CT/MRI) overall and by tumor type.<br>PK:<br>Plasma concentrations of onapristone, and mono-demethylated onapristone (M1) on day 1 at H0, 1, 2, 4, 6, 8, 12 (before next dose) and 24 (before next dose), and days 8, 29 and 57 at H0.<br>Plasma concentrations of di-demethylated onapristone (M2) and other metabolites on day 1.<br>Additionally, if a Grade 3-4 LFT elevation occurs, a PK sample will be drawn as soon as possible. This applies to the entire

	treatment period, even outside of the 8 week safety observation period.<br>In addition, urine will be analyzed for onapristone metabolites on day 1 and if G3-4 LFT elevation occurs.<br>Standard PK methods will be used.<br><ins>Exploratory analyses:</ins><br>CTCs will be enumerated and analyzed for PR status at baseline and every 4-8 weeks until progression in whole blood samples.<br>Plasma will be frozen at baseline for exploratory exosome analysis for gene signature determination
**Statistical Methods and Planned Analyses:**	<ins>Safety Analysis Population:</ins><br>The safety analysis population is defined as all patients who receive at least one dose of onapristone. The safety population will be used for all clinical data summaries.<br>Efficacy Analysis Population:<br>The efficacy analysis population is defined as all patients who have evaluable screening/baseline and at least one post-treatment tumor assessment.<br><ins>PK Analysis Population:</ins><br>The PK analysis population is defined as all patients who are evaluable for PK.<br><ins>Sample Size Justification:</ins><br>Pragmatic rule-based design to support dose selection and further explore safety in multiple tumor types at the recommended phase 2 dose.<br><ins>Safety:</ins><br>AEs/SAEs will be coded and tabulated using the current version of MedDRA.<br>Laboratory results will be tabulated in listings.<br>Concomitant medications will be coded using WHO Drug and tabulated.<br>Expectedness of the AEs will be assessed against the current Onapristone IB 2013.

2. TABLE OF CONTENTS

Page

1. SYNOPSIS 6
2. TABLE OF CONTENTS 13
3. LIST OF ABBREVIATIONS........ 18
4. BACKGROUND AND RATIONALE 20
4.1. Onapristone........ 20
4.1.1. PR Isoforms and Activated PR (APR) 21
4.1.2. Pharmacokinetics 22
4.1.3. Metabolism 22
4.1.4. Previous Clinical Studies in Breast Cancer 22
4.1.4.1. Phase 2 Study: Onapristone in Tamoxifen-Resistant Breast Cancer 22
4.1.4.2. Phase 2 Study: Onapristone in Hormone-Na1ve Patients with Breast Cancer (First-line Therapy) 23
4.1.4.3. Phase 3 Study: Onapristone versus Megestrol Acetate........ 24
4.1.4.4. Termination of Previous Development Program 25
4.1.5. Data in Endometrial Cancer and Other PR-positive Tumors 25
4.2. Clinical use of Progestin Agonists in Cancers Expressing the Progesterone Receptor 26
4.2.1. Endometrial Cancer........ 26
4.2.2. Breast Cancer........ 27
4.3. Rationale for Conducting the Study........ 27
4.4. Benefit/Risk Aspects........ 28
5. STUDY OBJECTIVES AND ENDPOINTS 30
5.1. Objectives........ 30
5.1.1. Primary Objectives........ 30
5.1.2. Secondary Objectives........ 30
5.1.3. Exploratory Objectives........ 30
5.2. Endpoints........ 30
5.2.1. Primary Endpoints........ 30
5.2.2. Secondary Endpoints........ 30

5.2.3. Exploratory Endpoints 31
6. INVESTIGATIONAL PLAN 32
6.1. Description of Overall Study Design and Plan 32
6.2. Discussion of Study Design 33
7. STUDY POPULATION 35
7.1. Study Population 35
7.2. Inclusion Criteria 35
7.3. Exclusion Criteria 35
7.4. Removal of Patients from Study 37
7.5. Replacement of Patients in Study 38
7.6. Data Review Committee 38
8. TREATMENTS 39
8.1. Details of Study Treatment 39
8.2. Manufacture of Investigational Medical Product 39
8.2.1. Packaging and Labeling of Onapristone Drug Product 39
8.2.2. Conditions for Storage of Onapristone Drug Product 40
8.2.3. Accountability 40
8.3. Dosage Schedule 40
8.3.1. Dose Administration 40
8.3.2. Dose Modification 40
8.4. Treatment Assignment 41
8.5. Blinding 42
8.6. Treatment Compliance 42
8.7. Prior and Concomitant Illnesses and Medications 42
8.7.1. Prior and Concomitant Illnesses 42
8.7.2. Prior and Concomitant Medications 42
8.7.2.1. Prohibited Treatment 43
8.7.2.2. Other Restrictions 43
9. STUDY PROCEDURES 44
9.1. Screening: Day -21 to Day 0 44
9.2. Baseline: Day 1 44
9.3. Treatment period visits (until week 8) 45

9.4. Treatment period visits (after week 8) 46
9.5. Follow-up visit (30 days (+/- 2 days) after last dose) 46
10. DESCRIPTION OF ASSESSMENTS 48
10.1. Efficacy Assessments 48
10.1.1. Tumor Tissue or Biopsy 48
10.2. Pharmacokinetic Assessments 48
10.2.1. Blood Samples 49
10.2.2. Urine Samples 49
10.2.3. Sample Preparation 50
10.2.4. PK Sample Processing Procedures 50
10.2.5. PK Sample Storage Conditions 50
10.2.6. PK Sample Shipping Procedures 50
10.3. Blood for Circulating Tumor Cells 51
10.4. Plasma for Exosome Analysis 51
10.5. Safety Assessments 51
10.6. Demographics 51
10.7. Physical Examination 52
10.8. Vital Signs 52
10.9. Electrocardiograms 52
10.10. Laboratory Parameters 52
10.11. Medical history 53
10.12. Serology/virology 53
10.13. Adverse Events 53
10.13.1. Serious Adverse Events 55
10.13.2. Other Significant Adverse Events and Suspected, Unexpected Serious Adverse Reactions 56
10.14. Concomitant Medication 56
10.15. Total Blood Volume Taken in Study 57
10.16. Study Information Card 57
11. DATA MANAGEMENT AND STATISTICAL ANALYSIS 58
11.1. Sample Size Justification 58
11.2. Sampling Schedule 58

11.3. Safety Population ... 58
11.4. Efficacy Analysis Population ... 58
11.5. PK Analysis Population ... 58
11.6. Patient Disposition and Termination Status ... 59
11.7. Background and Demographic Characteristics ... 59
11.8. Primary Endpoints ... 59
11.9. Secondary Endpoints ... 59
11.10. Exploratory Endpoints (CTC and Exosomes) ... 59
11.11. PK Analysis ... 60
11.12. Safety Evaluation ... 60
11.12.1. Adverse Events ... 61
11.12.2. Clinical Laboratory Evaluation ... 61
11.12.3. Physical Examination ... 61
11.12.4. Vital Signs ... 61
11.12.5. 12-Lead ECG ... 61
11.12.6. Physical Examination ... 62
11.12.7. Prior and Concomitant Medications ... 62
11.13. Efficacy ... 62
11.14. Protocol Deviations ... 62
12. STUDY MANAGEMENT ... 63
12.1. Approval and Consent ... 63
12.1.1. Regulatory Guidelines ... 63
12.1.2. CPP/Independent Ethics Committee ... 63
12.1.3. Informed Consent ... 63
12.2. Protocol Amendments and Other Changes in Study Conduct ... 63
12.3. Discontinuation of the Study by the Sponsor ... 64
12.4. Study Documentation ... 64
12.5. Data Handling ... 64
12.6. Study Monitoring and Auditing ... 65
12.7. Retention of Records ... 65
12.8. Use of Study Findings ... 66
12.9. Publication ... 66

13. REFERENCES 67

14. APPENDICES 71

APPENDIX 1. SCHEDULE OF ASSESSMENTS 72

APPENDIX 2. SCHEDULE OF BLOOD AND URINE SAMPLING FOR PHARMACOKINETICS (INCLUDING TOTAL BLOOD VOLUMES IN COMBINATION WITH SAFETY LABORATORY BLOOD DRAWS) 74

APPENDIX 3. DECLARATION OF HELSINKI 75

APPENDIX 4. SUBSTRATES OF CYP450 3A4 80

APPENDIX 5. HY'S LAW 81

APPENDIX 6. TISSUE BLOCK GUIDELINES 82

APPENDIX 7. INSTRUCTIONS FOR PROCESSING ASCITIC OR PLEURAL FLUID TO PRODUCE A PELLET FOR IHC 83

APPENDIX 8. COCKROFT-GAULT EQUATION FOR CALCULATED CREATININE CLEARANCE 84

LIST OF TABLES

Table 1: Details of Study Treatment 39

Table 2: Dose Modification Table 41

Table 3: Classification of Adverse Events by Intensity 54

Table 4: Classification of Adverse Events by Relationship to Study Medication 55

LIST OF FIGURES

Figure 1: Proposed Model of Subnuclear Distribution of PR Isoforms [Arnett Mansfield 2007] 20

Figure 2: PR Nuclear Foci Pattern in Endometrial Cancer Utilizing Antibodies to (A) PRA and to (B) PRB 21

Figure 3: Structure of Onapristone (ONA), N-Desmethyl Onapristone (M1, Mono-Demethylated Onapristone) and di-demethylated onapristone (M2) 22

Figure 4: Effect of Onapristone in an ER-positive, PR-indeterminate Xenograft Prevention Model of Endometrial Carcinoma 25

Figure 5: Study Design 33

3. LIST OF ABBREVIATIONS

AE	adverse event
ALT (SGPT)	alanine aminotransferase (serum glutamic pyruvic transaminase)
ANC	absolute neutrophil count
APR	activated progesterone receptor
AST (SGOT)	aspartate aminotransferase (serum glutamic oxaloacetic transaminase)
AUC_{0-last}	area under the plasma-concentration time curve from time 0 to the last quantifiable concentration
AUC_{0-oo}	area under the plasma-concentration time curve from time 0 to infinity
BID	twice daily
BMI	body mass index
BPM	beats per minute
^{11}C	carbon 11 radiolabel
CBC	complete blood cell count
C_{max}	maximum observed plasma concentration
CRF	case report form
CPP	Comite de Protection des Personnes (Ethics Committee)
CR	complete remission
CRA	clinical research associate
CSR	clinical study report
CT	computed tomography
CTCAE	Common Terminology Criteria for Adverse Events
CTCs	circulating tumor cells
CTM	clinical trial manager
CyP450 3A4	cytochrome P 450 3A4
DCIS	ductal carcinoma in situ
DLT	dose-limiting toxicity
DNA	deoxyribonucleic acid
ECG	electrocardiogram
ECOG	Eastern Cooperative Oncology Group
EMA	European Medicines Agency
ER	estrogen receptor
FDA	Food and Drug Administration
GCP	good clinical practice
GGT	y-glutamyl transpeptidase
GLP	Good Laboratory Practice
GMP	Good Manufacturing Practice
H	hour
hPR	human progesterone receptor
IB	Investigator's Brochure
ICH	International Conference on Harmonisation
IEC	independent ethics committee
IRB	institutional review board
IV	intravenous

LDH	lactate dehydrogenase
LFTs	liver function tests
MCV	mean corpuscular volume
MedDRA	Medical Dictionary for Regulatory Activities
MPA	medroxyprogesterone acetate
MRI	magnetic resonance imaging
NCA	non-compartmental analysis
PCOS	polycystic ovarian syndrome
PD	progressive disease or pharmacodynamic(s)
PK	pharmacokinetic(s)
PR	partial remission, progesterone receptor
PT	prothrombin time
PTT	partial thromboplastin time
QD	once daily
QRS	complex on ECG
QT, QTc	interval between Q and T wave on ECG
RECIST	Response Evaluation Criteria in Solid Tumors (Eisenhauer 2009)
RP2D	recommended phase 2 dose
SAE	serious adverse event
SAP	statistical analysis plan
SD	stable disease
SOC	system organ class
SOPs	standard operating procedures
SUSAR	suspected unexpected serious adverse reaction
$t_{1/2}$	elimination half-life
TEAE	treatment-emergent adverse event
T_{max}	time of maximum observed plasma concentration
ULN	upper limit of normal
UPLC-MS/MS	Ultra-high-performance liquid chromatography tandem mass spectrometry
V_c	volume of distribution in the plasma (central) compartment
VEGF	vascular endothelial growth factor
Az	terminal elimination rate constant
WBC	white blood cell
WHO	World Health Organization

4. BACKGROUND AND RATIONALE

4.l. Onapristone

Onapristone is a type I progesterone antagonist, which prevents the progesterone receptor (PR) monomers from dimerizing, inhibits ligand-induced phosphorylation of the PR and prevents association of the PR with its co-activators, thus preventing PR-induced DNA transcription (See Figure 1).

Figure l: Proposed Model of Subnuclear Distribution of PR Isoforms [Arnett Mansfield 2007]

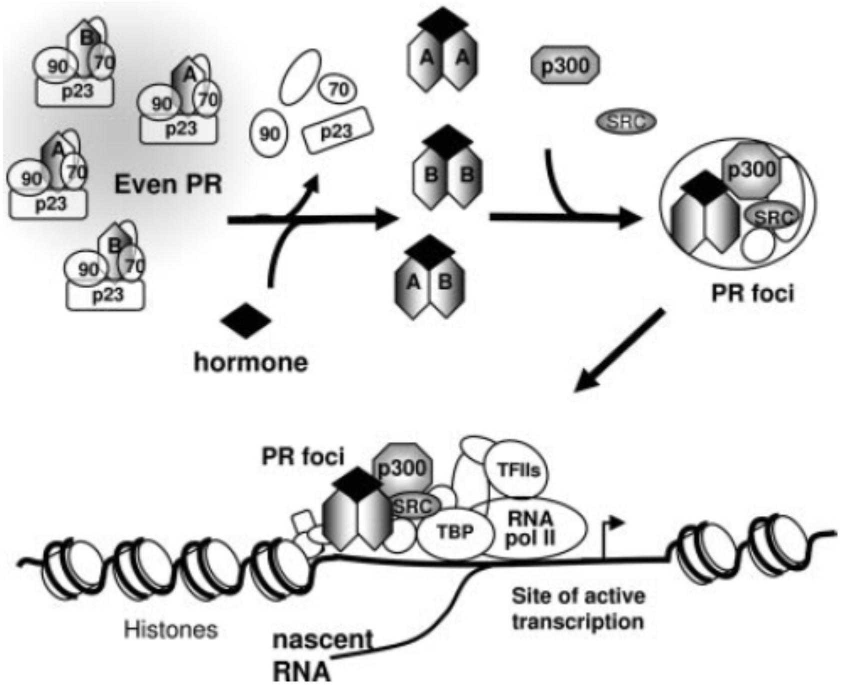

Onapristone prevents the PR from dimerizing [Allan 1992] and does not result in ligand induced phosphorylation of the PR [Beck 1996, Clemm 2000]; non-dimerized PR does not form nuclear PR foci and does not bind to DNA [Graham 2009]. This aspect is key to the activity of onapristone, as dimerization is necessary for binding with the coactivators and subsequently to the specific PR DNA responsive elements. Without functional PR dimers, no DNA transcription occurs. Studies have been conducted to examine the ability of PR to recruit a chromatin remodeling complex to the promoter when bound to different classes of antagonists and the influence of this interaction on PR dynamics and function in vivo. Onapristone, as opposed to other ligands, does not induce chromatin-remodeling, attachment of PR complex to DNA and recruitment of co-factors [Arnett-Mansfield 2007]. Onapristone has also been demonstrated to disassemble ligand-induced PR-DNA complexes [Rayasam, 2005].

ARN-AR18-CT-101 Page 2l of 84

[Redacted]

5. STUDY OBJECTIVES AND ENDPOINTS

5.l. Objectives

5.l.l. Primary Objectives

The primary objective of this study is to determine the RP2D of a single agent extended-release tablet formulation of oral onapristone for future clinical development.

5.l.2. Secondary Objectives

The secondary objectives of this study are to:

- Determine the safety profile of extended-release oral onapristone tablets administered on a chronic BID schedule.
- Determine the safety profile of an oral 100 mg immediate-release tablet formulation of onapristone on a chronic QD schedule.
- Compare the safety of the extended-release tablets BID and immediate-release tablets QD.
- Study the PK of chronic daily dosing of the extended-release and immediate-release onapristone tablet formulations.
- Evaluate potential anti-cancer efficacy at each dose level.

5.l.3. Exploratory Objectives

The exploratory objectives of this study are:

- Enumeration and testing of PR in circulating tumor cells (CTCs) to inform companion diagnostic development.
- Exosome analysis for gene signature determination.

5.2. Endpoints

5.2.l. Primary Endpoints

- Determination of the RP2D of oral extended-release onapristone tablets utilizing a day 57 safety cut off.

5.2.2. Secondary Endpoints

- Safety and tolerability of extended-release onapristone tablets BID and of immediate-release onapristone tablets QD.
- Comparison of safety of extended-release BID vs. immediate-release QD schedules.
- Efficacy based on tumor assessments (RECIST 1.1) and dates of progression.

- PK of onapristone, mono-demethylated onapristone and other metabolites in plasma and urine.

5.2.3. Exploratory Endpoints

- Level of progesterone receptors in circulating tumor cells.
- Exosome analysis of plasma for gene signature determination.

6. INVESTIGATIONAL PLAN

This investigational plan describes the study design, methods and the time course of the examinations in the planned study. It ensures consistency between conduct of the study, regulatory requirements and other guidance for clinical studies, ethical principles of the Declaration of Helsinki (Appendix 3), and the Standard Operating Procedures (SOPs) valid for Arno Therapeutics, Inc.

6.l. Description of Overall Study Design and Plan

This is a multi-center, open-label, randomized, parallel group two-stage phase 1 study with an expansion component.

Stage l: Six dose cohorts using the extended release formulation (10 mg BID, 20 mg BID, 30 mg BID, 40 mg BID, 50 mg BID) and the immediate-release formulation 100 mg QD will be randomized in parallel. Six patients are planned to be enrolled in each cohort. Data review will be undertaken in an ongoing manner by the medical monitor. The data review committee will review the safety, PK and PD data after the first patient at each dose level has completed 4 weeks of therapy, after the 2nd patient at each dose level has completed 4 weeks of therapy and after the 3rd patient at each dose level has completed 4 weeks of therapy. In addition the data review committee will be convened to review any DLTs. If there are 2 or more DLTs in any cohort, that cohort will stop enrolling.

DLT is defined as confirmed grade □3 AE. If there is :S1 DLT in any cohort, that cohort will continue enrolling up to 6 patients. If any of the LFT tests is elevated to grade □3, the medical monitor will immediately assess the complete LFT profile and other safety data, discuss it with the investigator and the event will be classified as an isolated laboratory abnormality or a liver DLT AE. For example, an isolated grade 3 AST elevation together with a grade 2 ALT elevation may not be considered a DLT, but requires repeat examination and continued monitoring per protocol. All liver function abnormalities will be reviewed by the data review committee.

If DLTs are not observed at the highest dose level of extended-release onapristone and efficacy is not observed up to that dose level, the protocol may be amended to further escalate the dose.

Stage 2: The data review committee will be convened when 3 patients have been treated for 8 weeks at each dose level. This data review will be conducted to assess safety, efficacy and PK, with the intent of recommending a dose level to enroll additional patients. Once the RP2D has been determined, that dose cohort will be expanded by enrollment of an additional 20 patients, to a total of 26 patients to confirm the safety profile. If additional data beyond the first 3 patients at each dose level is required, further data review committee meetings will be convened until a RP2D can be recommended.

In all stages and for all patients, LFTs will be monitored weekly for the first 8 weeks, then every 2 weeks. Tumor assessments will be performed every 8 weeks and circulating tumor cells will be monitored. Safety assessment of each dose level will use a cutoff at day 57 (8

weeks of treatment) for determination of the RP2D. PK samples will be taken on days 1, 8, 29 and 57 to provide adequate data in at least 3 consenting patients at each dose level.

Treatment will continue until occurrence of an intolerable safety issue not addressable by dose interruption and reduction, or treatment failure, or if the patient elects to withdraw, or for non-compliance with either protocol-specified evaluations or onapristone treatment.

Figure 5: Study Design

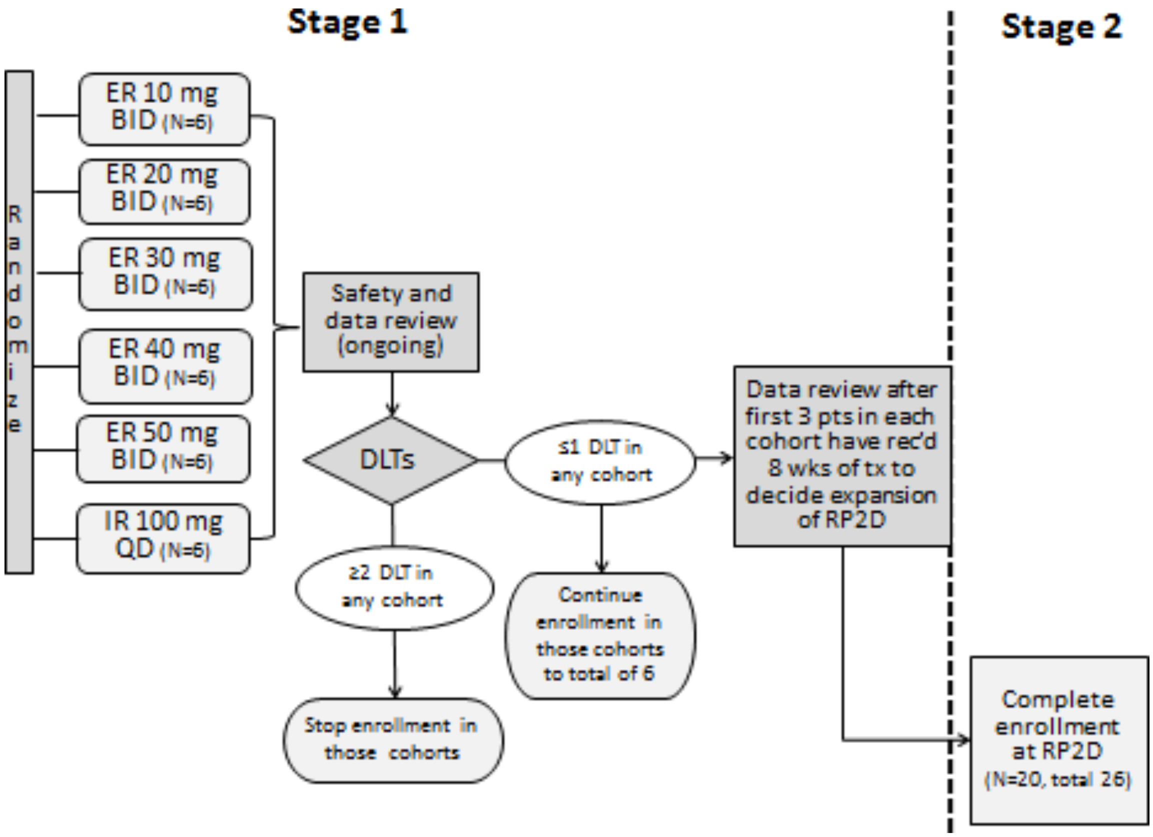

6.2. Discussion of Study Design

Stage 1: A randomized design with parallel dosing is justified since a higher dose than planned in this study had been given in prior studies with an acceptable safety profile by today's standards (100 mg daily or a maximal single dose of 400 mg). It is not known whether the same degree of LFT elevation will be seen at all dose levels, and therefore an efficacious but safe dose could inadvertently be eliminated in a sequential design.

The safety profile of each patient will be reviewed on an ongoing basis by the medical monitor, and the frequent planned reviews by the data review committee are intended to ensure the safety of individual patients in the context of the parallel dosing design.

Extended-release dosing twice a day justifies testing lower doses than previously shown to be effective in breast cancer (i.e. less than 100 mg/day). In addition it is not clear that a 100 mg dose is required to maximize the therapeutic efficacy of onapristone. Any efficacy data along

with the safety and PK data may demonstrate that a lower dose may be appropriate for the treatment of PR positive tumors.

Stage 2: This stage allows, in a controlled setting, a further understanding of the safety and tolerability whilst permitting preliminary efficacy signal-seeking in multiple tumor types.

All patients entered into the study are required to be PR positive. This will also provide the opportunity to evaluate the APR and correlate this to safety and any observed anti-tumor effect, thereby supplementing the knowledge base for the development of a predictive biomarker for response. All patients will provide an appropriate tissue sample for this part of the study (see Appendix 6).

Two dose reductions (to 75% and 50% of original dose) will be permitted for patients where continuation of therapy is thought to be in the patient's best interest, with the lowest permitted onapristone dose being 5 mg BID.

7. STUDY POPULATION

7.l. Study Population

The patient population for enrollment in this study will be female patients older than 18 years of age with cancer expressing the progesterone receptor.

7.2. Inclusion Criteria

Patients meeting all of the following inclusion criteria at screening/day-1 of first dosing will be eligible for participation in the study.

1. Post- menopausal female patients, 18 years of age or greater. Definition of post-menopausal: 6 month absence of regular menstrual cycles in the absence of endocrine manipulation or pregnancy/lactation or absence of ovarian function.
2. Recurrent or metastatic PR-expressing cancer which has the potential to benefit from an anti-progestin treatment including but not limited to endometrial cancer, ovarian, or breast cancer or uterine sarcoma.
3. Patients who have metastatic or recurrent disease after previous surgery, radiation therapy, and/or chemotherapy are eligible. No restriction is placed on the number of prior therapies.
4. Evaluable disease per RECIST 1.1.
5. Appropriate archival AND current tissue blocks or biopsy specimens to determine ER/PR and APR status (see Appendix 6). An appropriate tissue sample is one which gives a reasonable chance of reflecting the PR status of the tumor.
6. Signed, written informed consent must be obtained and documented according to ICH-GCP, the local regulatory requirements, and local data protection laws prior to study-specific screening procedures.
7. ECOG performance status 0-1.
8. Health care coverage.

7.3. Exclusion Criteria

Patients meeting any of the following exclusion criteria at Screening/day -1 of first dosing will not be enrolled in the study.

1. Calculated creatinine clearance of <60 mL/min (based on the Cockcroft-Gault equation - see Appendix 8).
2. Patients with any other prior malignancy are not allowed except for the following:
   - Adequately treated basal cell or squamous cell skin cancer
   - In situ cervical cancer

- Adequately treated Stage I or II cancer from which the patient is currently in complete remission or other cancer from which the patient has been disease-free for 2 years

3. Body mass index (BMI) <18.5 or >35 kg/m^2.
4. On ECG a QTc(F) interval >480 msec or any clinically significant cardiac rhythm abnormalities.
5. Liver function tests documented within the screening period and on day -1 of treatment period:
   - Total bilirubin > ULN (except in patients diagnosed with Gilbert's disease).
   - Alkaline phosphatase > UNL or > 2.5 x UNL in case of liver metastases, or > 5 x UNL in case of bone metastases.
   - ALT/AST > UNL or > 2.5 x UNL in case of liver metastases.
6. Known positive virology/serology for human immunodeficiency virus (HIV)-1, HIV-2, hepatitis B (surface antigen), or hepatitis C (testing not required).
7. Chronic inflammatory liver condition. History or clinical evidence of any liver or biliary pathology including cirrhosis, infectious disease, inflammatory conditions, steatosis, or cholangitis (including ascending cholangitis, primary sclerosing cholangitis, obstruction, perforation, fistula of biliary tract, spasm of sphincter of Oddi, biliary cyst or biliary atresia).
8. Chronic adrenal failure or is receiving concurrent long-term corticosteroid therapy.
9. History or clinical evidence of any surgical or medical condition which the investigator judges as likely to interfere with the results of the study or pose an additional risk in participating e.g., active or clinically significant history of disease involving a major organ system-vascular, cardiac, uncontrolled hypertension, pulmonary, gastrointestinal, gynecologic, hematologic, neurologic, neoplastic, renal, endocrine or immunodeficiency, or clinically significant active psychiatric disorders.
10. Used any prescription medication during the prior 1 month that the investigator judges is likely to interfere with the study or to pose an additional risk to the patient in participating, specifically inhibitors or inducers of CYP3A4 (see Appendix 4).
11. Received an investigational product or been treated with an investigational device within 30 days prior to first drug administration, or plans to start any other investigational product or device study within 30 days after last drug administration.
12. Received prior systemic anticancer treatment (chemotherapy, targeted therapies including kinases inhibitors, antibodies, etc.) less than 5 half-lives before the first dose of study drug or radiotherapy within 30 days; toxicity of the anticancer treatment must have recovered to grade 1 or less.
13. Current progestin-based hormone replacement therapy. If a patient is on progestins, including hormone replacement therapy and natural products known to contain progestins, they must be stopped 7 days prior to beginning study treatment.

14. Lack of physical integrity of the upper gastrointestinal tract, malabsorption syndrome, or inability to swallow pills.
15. Has a mental incapacity or language barriers precluding adequate understanding, co-operation, and compliance with the study requirements.
16. Is, in the judgment of the investigator, unable or unwilling to comply with the requirements of the study.
17. Uncontrolled brain metastases or treatment by neurosurgical resection or brain biopsy within 4 weeks prior to Day 1.

7.4. Removal of Patients from Study

A patient will be considered to have completed the study in terms of evaluability for safety when she has completed 8 weeks of treatment, or discontinued prior to that for safety reasons.

The patient has the right to withdraw from the study at any time for any reason, without the need to justify her withdrawal.

The patient may be withdrawn from the study at the discretion of the investigator due to safety concerns or if non-compliant with study procedures to an extent judged to affect the conclusion of the study.

A termination case report form (CRF) page should be completed for every patient who received study medication, whether or not the patient completes the study. The reason for any early discontinuation should be indicated on this form.

If the reason for withdrawal is an AE, the specific event or the main laboratory abnormality(ies) will be recorded in the CRF. All patients who withdraw because of AEs will be followed up at suitable intervals in order to evaluate the course of the AE and to ensure reversibility or stabilization. The subsequent outcomes of these events will be recorded in the CRFs as well as the reasons for withdrawal.

The primary reason for a patient withdrawing prematurely should be selected from the following standard categories of early termination:

- *Adverse Event (AE)*: Clinical or laboratory events occurring that in the medical judgment of the investigator for the best interest of the patient are grounds for discontinuation. This includes serious and non-serious AEs regardless of relation to study drug.
- *Death*: The patient died.
- *Withdrawal of Consent*: The patient desired to withdraw from further participation in the study in the absence of an investigator-determined medical need to withdraw. If the patient gave a reason for withdrawing, it should be recorded in the CRF.
- *Protocol Violation*: The patient's findings or conduct failed to meet the protocol entry criteria or failed to adhere to the protocol requirements (e.g. study drug

noncompliance, failure to return for defined number of visits). The violation necessitated premature termination from the study.

- *Lost to Follow-Up*: The patient stopped coming for visits, and study personnel were unable to contact the patient.
- *Termination of the Study by the Sponsor for Any Reason*.
- *Other*: The patient was withdrawn for a reason other than those listed above. In this case the reason should be specified.

Any patients who discontinue will have safety measurements performed before discharge, if possible. In addition, they will be given a complete follow-up examination including a physical examination, vital signs, ECG, and safety laboratory tests (clinical chemistry, liver function, hematology, clotting and urinalysis) on a follow-up visit, if possible.

7.5. Replacement of Patients in Study

All patient data collected will be analyzed. If patients are not evaluable for safety, or if insufficient PK or PD data is available at a given dose level, a patient may be replaced. The additional patient(s) included to replace non-evaluable patients will be given new, unique patient IDs.

7.6. Data Review Committee

The data review committee will consist of 2 independent, experienced oncology drug developers preferably with expertise in PR-positive tumors, an experienced pharmaceutical safety officer, a clinical pharmacologist, an investigator representative and a company representative.

8. TREATMENTS

8.1. Details of Study Treatment

The investigational medical product is onapristone, a PR antagonist. Dosing levels will be extended-release tablets 10 mg BID, 20 mg BID, 30 mg BID, 40 mg BID or 50 mg BID; or immediate-release tablets 100 mg QD.

Table 1: Details of Study Treatment

Parameter	Preparation to be Administered
Drug Designation	(11β,13α,17α)-11-[4-(Dimethylamino)phenyl]-17-hydroxy-17-(3-fluoropropyl)estra-4,9-dien-3-one (onapristone)
Trade Name	To be determined
Manufacturer	Patheon Ltd, Covingham, Swindon, UK
Labeling	Theorem Clinical Services, Bad Soden, Germany
Dosage strength	Extended-release onapristone 2.5mg, 5mg, 10mg, 20mg; Immediate-release onapristone 25mg
Route	Oral
Formulation	Tablet

All investigational drug supplies will be provided by the sponsor.

8.2. Manufacture of Investigational Medical Product

All packaging and labeling operations will be performed according to Good Manufacturing Practice (GMP) and GCP guidelines, as well as the national regulatory requirements which apply to this clinical investigation.

Copies of the QP release certification for the packaging and labeling operations as well as certificates of analysis will be supplied to Biotrial and/or the investigative sites by Arno Therapeutics, Inc.

The authorized pharmacist must complete and return to Arno Therapeutics, Inc. the Drug Delivery Note verifying receipt of the study drug.

The contact person with respect to study medication is Dr. Alex Zukiwski (see List of Contacts).

8.2.1. Packaging and Labeling of Onapristone Drug Product

Onapristone drug product will be packaged in bottles containing 30 tablets per bottle. Each bottle will contain only one dosage strength.

Both the outer box and the individual bottles will have labels displaying the necessary information per ICH guidelines and EU/French law, to include:

- Number of XX mg Onapristone Tablets for Oral Use Only
- Lot No.: XXXXXX Retest date: XX/XXXX

- For biomedical research only
- EudraCT : 2013!002859!14
- Principal Investigator:
- Protocol: ARN-AR18-CT-101
- Storage instructions
- Sponsor: Arno Therapeutics, Inc., Flemington, NJ, USA; Telephone +1 862 703 7170

8.2.2. Conditions for Storage of Onapristone Drug Product

Onapristone drug product must be stored at controlled room temperature, between 20-25°C.

The investigator will ensure that the investigational medicinal product will be stored in appropriate conditions in a secure location with controlled access. The temperature in the locked area should be monitored daily and the values recorded.

8.2.3. Accountability

Bottles of oral onapristone extended-release tablets 2.5 mg, 5 mg, 10 mg, 20 mg and onapristone immediate-release tablets 25 mg will be supplied to the study sites.

Any unused onapristone drug product will be accounted for and destroyed by the study site per the site's own SOPs following reconciliation by the study monitor. Certificates of destruction will be returned to Arno Therapeutics, Inc.

8.3. Dosage Schedule

8.3.l. Dose Administration

Onapristone will be taken orally with 240 mL of water on an empty stomach, 2 hours before or after meals. Dosing of onapristone will take place in the morning for all dose levels and approximately 12 hours later for all the BID dosing levels.

8.3.2. Dose Modification

Dose modifications will be allowed in the case of grade 3 or grade 4 non-hematologic AEs (except for alopecia, which does not warrant treatment modification) according to Table 2. Two 25% dose reductions (to 75% and 50% of original dose) will be permitted for patients where continuation of therapy is thought to be in the patient's best interest, with the lowest permitted dose being 5 mg BID. If that is not effective in reducing the adverse events to a :S grade 2 level, treatment must be discontinued.

Nausea and/or vomiting should be controlled with adequate antiemetics. If grade 3 or 4 nausea/vomiting occurs in spite of antiemetics, the dose should be reduced by 25%. If tolerated, increase the onapristone back to the 100% dose.

If onapristone is withheld because of hepatic toxicity, including elevation of LFTs, LFTs must return to :S grade 1 within 2 weeks or treatment will be discontinued. Onapristone treatment will be discontinued in an individual patient if there is evidence of Hy's Law (see

Appendix 5). Additionally, if a Grade 3-4 LFT elevation occurs, a PK sample will be drawn as soon as possible. This applies to the entire treatment period, even outside of the 8 week safety observation period.

Onapristone should be discontinued in the event of disease progression.

Table 2: Dose Modification Table

Onapristone BID Dose	Starting Dose Tablets	First dose reduction 25%		Second dose Reduction 25%	
		BID Dose	Tablets	BID Dose	Tablets
10 mg	1 X 10 mg	7.5 mg	1 X 5 mg + 1 X 2.5 mg	5 mg	1 X 5 mg
20 mg	1 X 20 mg	15 mg	1 X 10 mg + 1 X 5 mg	10 mg	1 X 10 mg
30 mg	1 X 20 + 1 X 10 mg	22.5 mg	1 X 20 mg + 1 X 2.5 mg	15 mg	1 X 10 mg + 1 X 5 mg
40 mg	2 X 20 mg	30 mg	1 X 20 + 1 X 10 mg	20 mg	1 X 20 mg
50 mg	2 X 20 mg + 1 X 10 mg	37.5 mg	3 X 10 mg+ 1 X 5 mg+ 1 X 2.5 mg	25 mg	1 X 20 mg + 1 X 5 mg
**Onapristone QD Dose**	**Starting Dose Tablets**	**First dose reduction 25%**		**Second dose Reduction 25%**	
		**QD Dose**	**Tablets**	**QD Dose**	**Tablets**
100 mg	4 X 25mg	75 mg	3 X 25mg	50 mg	2 X 25mg

In the case of grade 3 or 4 hematologic AEs, treatment should be interrupted and the medical monitor should be consulted.

For Grade 1 and 2 toxicities, no dose reduction should be made.

8.4. Treatment Assignment

Patients will be randomly assigned to an onapristone dosage level using a computer-generated randomization list.

All screened patients will be given a unique identification number. Patients failing screening will be replaced until the specified number of eligible patients has been allocated to each part of the study and completed all study evaluations. Identification numbers assigned to ineligible or non-evaluable patients will not be re-used.

Throughout the study, unique patient identification numbers will be assigned during screening and subsequently a randomization number will be also assigned at inclusion (different from the identification number assigned at screening).

At the screening visit the patients will be assigned a unique eight digit number composed of:

- The site code (4 digits, i.e., 0001)
- The patient code (4 digits in chronological order according to the number of screened patients at a given site, i.e. 0001, 0002, etc)

For example, the first patient at site 2 will be assigned the number 0002-0001.

The patient randomization number will be a 4-digit number provided by the study statistician, determined by:

- The randomization code (4 digits in chronological order according to the number of included patients across the entire study).

8.5. Blinding

Not applicable; this is an open-label study.

8.6. Treatment Compliance

Study drug will be provided by the Sponsor and receipt acknowledged by the study site.

The investigator is responsible for the control of drugs under investigation. Records of the receipt and disposition of the study drug must be maintained. The Drug Dispensing Log should contain the following information:

- identification of the patient to whom the study drug was dispensed
- date(s) and quantity of the study drug dispensed to the patient
- date(s) and quantity of the study drug returned by the patient
- records and drug supplies must be available for inspection by the study monitor

Patients will be asked to record the doses taken in a drug diary.

Study drug will either be disposed of at the study site according to the study site's institutional standard operating procedure or returned to the Sponsor with the appropriate documentation. The site's method of investigational medical product (IMP) destruction must be agreed with the Sponsor.

The site must obtain written authorization from the Sponsor before any IMP is destroyed, and IMP destruction must be documented on an appropriate form.

8.7. Prior and Concomitant Illnesses and Medications

8.7.l. Prior and Concomitant Illnesses

The investigator should document all prior significant illnesses that the patient has experienced within 1 year prior to screening. Additional illnesses present at the time of informed consent are to be regarded as concomitant illnesses. Illnesses first occurring or detected during the study and/or worsening of a concomitant illness during the study are to be documented as AEs on the CRF.

8.7.2. Prior and Concomitant Medications

Any concomitant medication used during the study or within the valid time range before dosing (two weeks or five half-lives of the drug, whichever is longer, prior to the dosing day) will be recorded in the CRF, together with the main reason for its prescription. Also the dose and dosage regimen will be documented in the CRF.

Particular care should be taken to document the concomitant use of inhibitors, inducers and substrates of CyP450 3A4 (see Appendix 4).

8.7.2.l. Prohibited Treatment

Patients should not receive other investigational agents or participate in a device study within 30 days prior to screening and will not start any other investigational product or device study within 30 days after last drug administration.

8.7.2.2. Other Restrictions

None.

9. STUDY PROCEDURES

The schedule of assessments (see Appendix 1 and Appendix 2) lists all assessments to be performed during the study. Unless otherwise specified, all assessments will be performed by the investigator or other study personnel. The assessments to be performed are outlined in the following by-visit subsections.

9.l. Screening: Day -2l to Day 0

Screening will occur within day-21 to Day 0 and the following assessments will be performed:

- Obtain signed informed consent.
- Record demographics.
- Record medical history.
- Complete physical examination, including height, weight, ECOG.
- Record vital signs.
- Conduct 12-lead ECG.
- Obtain blood samples for hematology, coagulation, chemistry and liver function.
- Obtain previous test results for serology / virology, if applicable.
- Obtain blood sample for serum cortisol levels.
- Obtain urine sample for urinalysis.
- Verify inclusion and exclusion criteria.
- Record prior and concomitant medications.
- Record AEs (following signed ICF).
- Verify PR status of the tumor.
- Perform CT/MRI for tumor assessment.
- Biopsy for tumor block if required (see Appendix 6).

9.2. Baseline: Day l

Baseline is the day of first study drug treatment, and the following procedures will be performed:

- Abbreviated (targeted) physical examination, including weight, ECOG.
- Record vital signs.
- Conduct 12-lead ECG.
- Obtain blood samples for hematology, coagulation, chemistry and liver function.

- Obtain blood samples for PK on day 1 at H0, 1, 2, 4, 6, 8, 12 (before next BID dose).
- Obtain blood sample for CTCs.
- Obtain blood sample for exosome analysis.
- Obtain urine for urinalysis.
- Obtain PK urine samples over the following time ranges: pre-dose, 0-1, 1-3, 3-6, 6-12, and 12-24 hours post-dose (Institut Curie patients only).
- Verify inclusion and exclusion criteria.
- Randomization.
- Record concomitant medications.
- Record AEs.

9.3. Treatment period visits (until week 8)

Patients will return to the clinic weekly until week 8, where the following procedures will be performed:

- Abbreviated (targeted) physical examination, including weight, ECOG (every 4 weeks).
- Record vital signs (every 2 weeks).
- Conduct 12-lead ECG (every 2 weeks).
- Obtain blood samples for hematology, coagulation, chemistry (every 4 weeks).
- Obtain blood samples for liver function (every week). Additionally, if a Grade 3-4 LFT elevation occurs, a PK blood sample will be drawn and urine collected as soon as possible.
- Obtain blood samples for PK on day 2 at H24 (before next dose) for 100 mg immediate-release formulation patients only, and days 8, 29 and 57 at H0 for all patients.
- Obtain blood sample for CTCs (every 4 weeks until PD).
- Obtain urine sample for urinalysis (every 4 weeks).
- On day 2 obtain PK urine sample over the range: 12-24 hours post-dose (Institut Curie patients only).
- Obtain blood sample for serum cortisol levels (week 4 only).
- Tumor assessment (week 8 only).
- Record concomitant medications (every week).
- Record AEs (every week).

Note: Every effort should be made to perform the post-day 1 study visits on the planned days. However, a window of +/- 1 day is acceptable during the initial 57 days of therapy.

9.4. Treatment period visits (after week 8)

Patients will return to the clinic every 2 weeks after week 8, where the following procedures will be performed:

- Abbreviated (targeted) physical examination, including weight, ECOG (every 4 weeks).
- Record vital signs (every 4 weeks).
- Conduct 12-lead ECG (every 4 weeks).
- Obtain blood samples for hematology, coagulation, chemistry (every 4 weeks).
- Obtain blood samples for liver function (every 2 weeks). Additionally, if a Grade 3-4 LFT elevation occurs, a PK sample will be drawn as soon as possible.
- Obtain blood sample for CTCs (every 8 weeks until PD).
- Obtain urine sample for urinalysis (every 4 weeks).
- Tumor assessment (every 8 weeks).
- Record concomitant medications (every 2 weeks).
- Record AEs (every 2 weeks).

Note: Every effort should be made to perform the study visits on the planned days. However, a window of +/- 2 days is acceptable after day 57.

9.5. Follow-up visit (30 days (+/- 2 days) after last dose)

Patients will return to the clinic 30 days after last administration of onapristone, for a final follow-up visit where the following procedures will be performed:

- Abbreviated (targeted) physical examination, including weight, ECOG.
- Record vital signs.
- Conduct 12-lead ECG.
- Obtain blood samples for hematology, coagulation, chemistry.
- Obtain blood samples for liver function. Additionally, if a Grade 3-4 LFT elevation occurs, a PK sample will be drawn as soon as possible.
- Obtain urine sample for urinalysis.
- Obtain blood sample for CTCs (non-progressing patients only).
- Tumor assessment (non-progressing patients only).
- Record concomitant medications.

- Record AEs.
- Discharge patient from study unless LFTs are elevated >10% above baseline, and this is attributable to onapristone, or another related AE is considered unresolved. In this case, patients will be followed up with weekly repeat LFTs until resolution to within 10% of baseline value.

10. DESCRIPTION OF ASSESSMENTS

10.1. Efficacy Assessments

Tumor assessments will be performed to assess disease response per RECIST 1.1 (CT/MRI).

10.1.1. Tumor Tissue or Biopsy

Pharmacodynamic assessments will include analysis of tumor biopsy samples for PR and APR receptor status.

The analysis can be performed on archived tumor blocks, or if these are not available a new tumor biopsy should be performed during screening. See Appendix 1, Appendix 6 and Appendix 7. Tissue obtained will be used in pharmacodynamic research studies proposed to investigate the presence of PR receptors, their subtypes and the extent of activation of those receptors. These studies will analyze the levels of PRA, PRB and APR.

The collection, storage, and shipping of tissue samples will be performed as detailed in a Study Procedures Manual, to be provided to study sites prior to study initiation.

The goal of the proposed biomarker study is to correlate the baseline types of PR and extent of APR to tumor response and dates of progression if possible. It is also intended to establish the basis for development of a companion diagnostic.

10.2. Pharmacokinetic Assessments

Quantitative measurement of plasma and urine concentrations of onapristone, mono-demethylated onapristone and other metabolites will be analyzed by the Radiopharmacology Department of Institut Curie, St Cloud, France using a validated UPLC method with MS/MS detection.

PK samples will be analyzed in expeditious fashion to permit the data review committee to make dose level decisions according to the results.

The method is validated according to the principles defined in applicable regulatory guidelines [EMA 2011, FDA 2001] and measurements will be carried out according to Good Laboratory Practice (GLP). After completion of the sample analyses, a bioanalytical study report will be issued and referenced in the clinical study report.

Venous blood samples (2.6 mL aliquots, all patients) and urine samples (Institut Curie patients) will be taken at the specific time points designated in the Blood and Urine Sampling Schedule (Appendix 2) and the Schedule of Assessments (Appendix 1).

Pharmacokinetic variables for drug (and, if required, any drug metabolites) will be calculated from the plasma concentration data using noncompartmental methods. The following parameters will be determined:

- C_{max}: Maximum observed plasma concentration
- T_{max}: The time that C_{max} is observed

- AUC_{0-t}: Area under the concentration-time curve from time zero to time (t)
- AUC_{0-1}: Area under the concentration-time curve from time zero to time (1); where tau is the length of the dosing interval
- AUC_{0-last}: Area under the concentration-time curve from time zero to the last quantifiable concentration
- AUC_{0-oo}: Area under the concentration-time curve from time zero to infinity; calculated as $AUC_{0-last} + C_{last}/Az$
- $t_{½}$: Terminal elimination half-life; calculated as 0.693/Az
- Az: The terminal elimination rate constant
- CL: Plasma clearance
- Vc: Volume of distribution in the plasma (central) compartment

All samples for pharmacokinetic analysis will be performed by the radiopharmacology department of the Institut Curie. The collection, storage, and transport of plasma and urine samples will be performed as described in a study procedures manual to be provided to study sites prior to study initiation.

10.2.1. Blood Samples

The blood samples (one 2.6 mL aliquots per timepoint) will be collected into 2.6 mL plastic citrate-containing tubes at the defined times stated in the Blood Sampling Schedule (Appendix 2) and the Schedules of Assessments (see Appendix 1). A separate Study Procedures Manual will provide greater detail on PK sampling, preparation, processing and storage at the study sites.

Blood collection schedule

In all dose levels, 8-9 blood samples will be collected for complete PK analysis on day 1 t0, 1h, 2h, 3h, 4h, 6h, 8h, and 12h (before second administration of extended-release formulation BID) and 24h (before second administration only for the immediate-release 100 mg). Trough levels will be collected at t0 on days 8, 29 and 57.

If possible, once the initial 18 patients have been accrued and treated, a limited sampling strategy will be proposed in order to decrease the number of blood samples to potentially as few as 4, using the data obtained from this complete PK analysis and the data obtained from the ongoing onapristone food effect study (ARN-AR-18-CT-001).

If a patient experiences LFT elevation, an attempt will be made to collect blood for PK analysis in a timely fashion.

10.2.2. Urine Samples

On day 1, each patient will empty their bladder just prior to onapristone administration. An aliquot of 5 mL of this "blank urine" will be labeled and used as a baseline control for the post-dose measurements.

After administration, urine will be collected according to the sampling time schedule below, for 24 hours. At the end of each collection period, the total volume will be measured and recorded. Each patient will be asked to empty their bladder at the end of each urine collection interval and this urine will be included in the total for that interval.

Urine collection schedule

In all patients treated at Institut Curie, urine will be collected for PK analysis on day 1 over the following time periods (fractions): pre-dose, 0-1, 1-3, 3-6, 6-12, and 12-24 hours post-dose, until sufficient information is collected to permit discontinuation of the urine PK study.

If a patient experiences LFT elevation, an attempt will be made to collect urine for PK analysis in a timely fashion.

l0.2.3. Sample Preparation

Blood samples will be chilled on wet ice immediately after collection into citrate-containing tubes.

Urine will be collected in standard urine collection containers.

l0.2.4. PK Sample Processing Procedures

Blood sample processing

Blood samples will be placed in wet ice immediately after collection and remain on ice until centrifugation, within 30 minutes of collection. Plasma will be separated by centrifugation (2,000g for 10 minutes) at approximately 4°C and resulting plasma will promptly be transferred to an appropriately-labeled polypropylene (1.8 or 3.6) ml NUNC tube.

Urine sample processing

All urine samples from each set time period (fraction) above will be thoroughly mixed together. After thorough mixing, an aliquot of 10 mL of each fraction will be labeled (nature of sample, time interval of void, volume, initials/identification number of the patient and protocol number). Urine sample tubes will be immediately placed upright in a -20 to -80°C freezer, where they will be stored until shipping.

Further processing instructions for PK samples are provided in the separate Study Procedures Manual.

l0.2.5. PK Sample Storage Conditions

All plasma and urine tubes must be stored upright in a -20 to -80 °C freezer. Individual specimen container labeling should include the study number, patient number; study period, formulation, study day, date and nominal sampling time.

No more than 30 minutes should elapse between sample collection and processing.

l0.2.6. PK Sample Shipping Procedures

Plasma and urine PK samples will be stored between -20 and -80 °C until transported for analysis to the Radiopharmacology Department at H6pital Rene Huguenin, Institut Curie (see

address page 5). The site will notify the Clinical Pharmacologist (Keyvan Resai; see page 5) prior to shipping and send an electronic sample manifest in Excel®.

The Radiopharmacology Department will receive a copy of the randomization schedule for this study prior to sample analysis.

Shipping address:

Radiopharmacology Department

Institut Curie-H6pital Rene-Huguenin

35, Rue Dailly

92210 St Cloud, France

World Courier will collect the samples from the 2 clinical sites on demand for delivery to the Institut Curie Radiopharmacology Department. For additional information on arranging sample pickup and sample handling, see separate Study Procedures Manual.

l0.3. Blood for Circulating Tumor Cells

10 mL of whole blood will be drawn into prespecified 10 mL tubes for central processing. Kits for collection and shipping instructions will be provided to the investigative sites.

l0.4. Plasma for Exosome Analysis

2.5 mL of blood will be drawn into PAXgene Blood RNA Tubes at baseline and frozen at -80°C for later analysis.

l0.5. Safety Assessments

Safety assessments will be performed at the intervals indicated in the Schedule of Assessments (see Appendix 1) and at any time deemed necessary by the investigator.

AEs and SAEs (including abnormal laboratory test results) will be collected from the time of signing the informed consent until 30 days after the last onapristone dose. Only AEs/abnormalities occurring after the first dose of onapristone will be considered treatment-emergent.

Weekly LFT labs should preferably be drawn at the study sites rather than at an external laboratory.

l0.6. Demographics

Information about date of birth, gender, race, detailed smoking history and alcohol history will be recorded during screening.

10.7. Physical Examination

Complete physical examinations will include examination of general appearance, skin, neck (including thyroid), eyes, nose, throat, heart, lungs, abdomen, lymph nodes, extremities, reproductive and nervous system and measurement of body weight.

Abbreviated (targeted) physical examinations should include a cardiorespiratory assessment and abdominal exam, but will focus on new symptoms and will include examination of relevant systems as identified by the investigator. An AE form should be completed for all changes identified as clinically significant.

Height and weight will be evaluated at screening and the BMI will be calculated.

10.8. Vital Signs

Vital signs will include body temperature, respiratory rate, radial pulse rates, and systolic and diastolic blood pressures.

Blood pressure and heart rate will be measured after 5 minutes in a semi-recumbent position by means of oscillometry, using an automatic blood pressure measuring device.

If systolic blood pressure is below 100 mmHg or above 140 mmHg and/or diastolic pressure is below 50 mmHg or above 90 mmHg, measurement will be repeated. The heart rate measurement will be repeated when below 50 beats per minutes (bpm) or above 90 bpm. If the measurement is still outside normal ranges, it is up to the investigator to judge if the measurement should be repeated.

Vital sign measurements outside normal ranges will be assessed as 'abnormal, not clinically significant', or 'abnormal, clinically significant' by the investigator. In the latter case, the abnormal vital sign measurement will be reported as an AE and further investigated as clinically indicated.

10.9. Electrocardiograms

Standard 12-lead ECGs will be conducted at Screening and at the same time as physical examinations. A single standard 12-lead ECG will be performed at each timepoint.

The ECG parameters to be documented in the CRF are as follows: rhythm, PR interval, heart rate, QRS interval, QT interval, Qtcii interval, and QRS axis.

ECGs will be recorded at bedside while the patient is resting in a supine or in a semi-recumbent position. ECGs will be categorized as 'normal', 'abnormal, not clinically significant', or 'abnormal, clinically significant'.

All ECGs will be printed, copied, de-identified and labeled with patients' unique study identifiers and are to be provided to Arno Therapeutics, Inc.

10.10. Laboratory Parameters

The following laboratory tests are to be performed as indicated by the Schedules of Assessments (see Appendix 1 and Appendix 2). All laboratory tests will be analyzed by the same local laboratory throughout the study, as designated by the principal investigator.

- Hematology: hemoglobin, hematocrit, red blood cell count, white blood cell count (with differential), platelet count, mean corpuscular volume (MCV) and absolute neutrophil count (ANC).
- Coagulation: prothrombin time, activated partial thromboplastin time, international normalized ratio (INR).
- Chemistry: creatinine, fasting blood glucose and fasting lipid profile, phosphate, total protein, sodium, chloride, magnesium, potassium, and calcium.
- Liver function: albumin, ALT, alkaline phosphatase, AST, bilirubin-direct, bilirubin-total, gamma GT, and LDH.
- Urinalysis (dipstick): pH, specific gravity, ketones, albumin, protein, glucose, bilirubin, and blood; in addition, a microscopic examination is to be performed if more than 1 test is positive for blood.

In the event of an unexplained clinically significant abnormal laboratory test value, the test should be repeated immediately and followed up until it has returned to the normal range and/or an adequate explanation of the abnormality is found. Particular vigilance should be exercised for liver function abnormalities and, if elevations attributable to onapristone occur, patients should be followed up until resolution to within 10% of absolute baseline values.

10.11. Medical history

Medical history will be recorded during screening to ensure eligibility of the patients and will focus on relevant current or past abnormalities or diseases of the following systems: gynecologic, cardiovascular, respiratory, gastrointestinal, hepatic, biliary, renal, endocrine/metabolic, musculoskeletal, hematologic/lymphatic, neurologic/psychiatric, dermatologic, immunologic, and infectious disease, bleeding tendency, and allergy/drug sensitivity.

10.12. Serology/virology

Serology and virology measurements are not required to be performed at screening but the results of the following previously performed serology tests should be recorded if available for eligibility purposes:

- Hepatitis B virus surface-antigen
- Hepatitis C virus antibody
- HIV-1/HIV-2 antibodies

10.13. Adverse Events

An AE is any symptom, physical sign, syndrome, or disease that either emerges during the study or, if present at Screening, worsens during the study, regardless of the suspected cause of the event. All medical and psychiatric conditions (except those related to the indication under study) present at Screening will be documented on the Prior Illnesses CRF page. Changes in these conditions and new symptoms, physical signs, syndromes, or diseases

should be noted on the AE CRF page during the rest of the study. Laboratory, Vital signs and ECG abnormalities should also be recorded as AEs.

AEs may be volunteered spontaneously by the patient, discovered as a result of general questioning by the study staff, or determined by physical examination. During each visit to the study clinic, the patient will be asked, "Have you experienced any problems since your last visit?" All AEs will be recorded on the CRF. For all AEs, the investigator must pursue and obtain information adequate both to determine the outcome of the AE and to assess if it meets the criteria for classification as an SAE requiring immediate notification. Follow-up of the AE, even after the date of therapy discontinuation, is required if the AE persists until the event resolves or stabilizes at a level acceptable to the investigator.

In order to avoid vague, ambiguous, or colloquial expressions, all AEs should be recorded in standard medical terminology rather than the patient's own words. Each AE will also be described in terms of duration, frequency, intensity, association with the study medication, assessment of possible causes, actions taken, and outcome, using choices given on the CRF. Whenever possible, intensity will be classified according to the criteria provided by the CTCAE v4.0). If the AE is not listed in the CTCAE v4.0, then the highest intensity level reached according to the scale in Table 3 will be assigned. Specific guidelines for classifying AEs by relationship to study medication are given in Table 4.

Table 3: Classification of Adverse Events by Intensity

Grade 1	Mild: An AE that is easily tolerated by the patient. It incurs only a minimum of discomfort, and does not influence ordinary daily tasks.
Grade 2	Moderate: An AE that is of sufficient severity to have a negative influence on ordinary daily tasks.
Grade 3	Severe: An AE that effectively hinders ordinary daily tasks, often requiring intervention.
Grade 4	Life-threatening or disabling: An AE that puts the patient's life at risk.
Grade 5	Death related to an AE.

Table 4: Classification of Adverse Events by Relationship to Study Medication

**UNRELATED**:	This category applies to those AEs that are clearly and incontrovertibly due to extraneous causes (disease, environment, etc.).
**UNLIKELY**:	This category applies to those AEs that are judged to be unrelated to the test drug, but for whi clinical state, environmental or toxic factors, or other modes of therapy administered to the patient; (3) it does not follow a known pattern of response to the test drug; or (4) it does not reappear or worsen when the drug is re-administered.
**POSSIBLY**:	This category applies to those AEs for which a connection with the test drug administration appears unlikely but cannot be ruled out with certainty. An AE may be considered possibly related if or when it meets 2 of the following criteria: (1) it follows a reasonable temporal sequence from administration of the drug; (2) it could not readily have been produced by the patient's clinical state, environmental or toxic factors, or other modes of therapy administered to the patient; or (3) it follows a known pattern of response to the test drug.
**PROBABLY**:	This category applies to those AEs that the investigator feels with a high degree of certainty a environmental or toxic factors, or other modes of therapy administered to the patient; (3) it disappears or decreases on cessation or reduction in dose (note that there are exceptions when an AE does not disappear upon discontinuation of the drug, yet drug-relatedness clearly exists; for example, as in bone marrow depression, fixed drug eruptions, or tardive dyskinesia); or (4) it follows a known pattern of response to the test drug.
**DEFINITELY**:	This category applies to those AEs that the investigator feels are incontrovertibly related to te environmental or toxic factors, or other modes of therapy administered to the patient; (3) it disappears or decreases on cessation or reduction in dose and recurs with re-exposure to drug (if rechallenge occurs); and (4) it follows a known pattern of response to the test drug.

When changes in the intensity of an AE occur, record the start date and stop date of the entire AE with the worst grade for the entire time period.

10.13.1. Serious Adverse Events

An SAE is defined as any AE that meets one or more of the following criteria:

- The event is fatal or life-threatening.
- The event is permanently disabling (incapacitating or interfering with the ability to resume usual life patterns).
- The event results in unplanned in-patient hospitalization or prolongation of an existing hospitalization.
- The event requires medical intervention of any kind in order to prevent any of the aforementioned outcomes.

An AE does not need to be severe in order to be classified as an SAE. For example, an overnight hospitalization for a diagnostic procedure must be reported as an SAE even though the occurrence is not medically serious. By the same token, a severe AE is not necessarily serious: for example, nausea of several hours' duration may be rated as severe but may not be considered serious.

An SAE occurring during the study or within 30 days of stopping the treatment must be reported to the study safety coordinator. **Any such SAE due to any cause, whether or not related to the study medication, must be reported within 24 hours of occurrence or when the investigator becomes aware of the event**. The investigator must send a

preliminary report of any such SAE to the study safety coordinator by fax within 24 hours using an SAE Report Form.

Safety Monitor:

ICON Pharmacovigilance
ICON Clinical Research GmbH
Heinrich-Hertz-Strasse 26
63225 Langen
Germany
+49 6103 904 217 - Fax Number
+49 6103 904 1460 - Phone Number

The event must also be recorded on the standard AE CRF page. Preliminary reports of SAEs must be followed by detailed descriptions later on, including clear anonymized photocopies of hospital case reports, consultant reports, autopsy reports, and other documents when requested and applicable. All photocopies should be redacted to remove patients' personal details and annotated with the patient's unique study identifiers. SAE reports must be made whether or not the investigator considers the event to be related to the investigational drug.

Appropriate remedial measures should be taken to treat the SAE, and the response to treatment should be recorded. Patients must be closely followed until sufficient information is obtained to indicate a return to normal status or until the event stabilizes at a level acceptable to the investigator. Clinical, laboratory, and diagnostic measures should be employed as needed in order to determine the etiology of the problem. The results will be reported promptly to the sponsor.

10.13.2. Other Significant Adverse Events and Suspected, Unexpected Serious Adverse Reactions

To ensure patient safety, the investigator should also notify the safety coordinator or medical advisor should any AE occur that is considered significant but does not meet criteria for an SAE, or that is considered unexpected. An unexpected AE is an AE that is not identified in nature, intensity, or frequency in the Onapristone IB 2013. Any suspected, unexpected serious adverse reaction (SUSAR) must be reported to the sponsor, the Institutional Review Boards (IRBs)/Independent Ethics Committees (IECs) and the regulatory authorities within the required timeframes. The medical advisor may choose to discontinue the patient from the study. In addition, any field monitor who notes a significant AE or medical condition while reviewing the CRFs or source documents at the site must immediately convey this information to the medical advisor.

10.14. Concomitant Medication

Information about concomitant medication will be recorded during screening and throughout the study.

10.15. Total Blood Volume Taken in Study

The total blood volume drawn from each patient during the initial 8 week study period is calculated to be approximately 185 mL (Appendix 2).

10.16. Study Information Card

During the first treatment period the patient will be provided with a study information card that should be carried at all times until completion of the follow-up visit. The study information card states the following:

- That they are participating in a clinical study.
- That they have received the investigational drug onapristone.
- The name and phone number of the investigator.

11. DATA MANAGEMENT AND STATISTICAL ANALYSIS

Electronic CRFs will allow all clinical parameters to be entered directly into a database within 48 hours of each study visit. The statistical analysis of the clinical data will be performed by the sponsor's representative, in coordination with the clinical investigational unit. Analysis of the PK data may be performed separately and entered into a separate database. Data from the clinical and PK databases will be integrated in the clinical study report.

Current versions of standard clinical pharmacology software will be used for the various PK analyses. Standard clinical pharmacology analytical software such as R and Monolix® will be used for estimation of onapristone PK and repeat dose modeling.

All data obtained either from the CRFs or from an external laboratory will be listed, and summary tables will be provided.

A separate statistical analysis plan (SAP) detailing statistical assessment (except for PK) for this protocol will be prepared. Pharmacokinetic analysis will be described in a separate document.

Any changes in the statistical methods described herein compared to the final SAP will be documented in the integrated clinical study report.

11.1. Sample Size Justification

Sample size (up to 60 patients) was derived by a pragmatic rule-based design to support dose selection and further explore safety in multiple tumor types at the recommended phase 2 dose.

11.2. Sampling Schedule

The sampling schedules should permit characterization of average concentrations during dosage intervals, as well as accumulation upon chronic dosing. The expected plasma $t_{1/2}$of onapristone is approximately 2-4 hours.

11.3. Safety Population

The safety population is defined as all patients who receive at least one dose of onapristone. The safety population will be used for all clinical data summaries.

11.4. Efficacy Analysis Population

The efficacy analysis population is defined as all patients who have evaluable screening/baseline and at least one post-treatment tumor assessment.

11.5. PK Analysis Population

The PK analysis population is defined as all patients who are evaluable for PK.

11.6. Patient Disposition and Termination Status

The number and percentage of enrolled versus screened patients will be summarized.

Early withdrawals and the reason for withdrawal will be tabulated. The number and percentage of patients who complete the study and who withdraw from the study will be documented.

If patients have missing or uninterpretable data for PK, the investigator or sponsor may enroll an additional patient to replace the missing information and maintain the planned sample size for the analysis.

11.7. Background and Demographic Characteristics

Patient baseline characteristics; including demographics, medical history, physical examination, ECG, and vital signs will be summarized descriptively. The descriptive statistics, including n (number of observations or sample size), mean, standard deviation and/or standard error, median, range (minimum-maximum) for numerical variables, and frequency and percentages for categorical variables, will be presented.

11.8. Primary Endpoints

The primary endpoint RP2D of oral extended-release onapristone will be determined by safety data, PK and potentially preliminary efficacy data utilizing a day 57 safety cut off.

11.9. Secondary Endpoints

- Safety and tolerability of oral extended-release BID and immediate-release onapristone QD will be tabulated as worst grade per patient by dose level and overall and based on the number and type of adverse events; clinical laboratory tests including clinical chemistry, liver function, hematology, clotting and urinalysis, vital signs, 12-lead ECG and physical examination.
- Safety of extended-release BID will be informally compared vs. immediate-release QD schedules, assessed as above.
- PK of onapristone and mono-demethylated onapristone will be analyzed by dose level and overall, and the immediate-release kinetics compared to the extended-release.
- Efficacy assessment will be based on tumor assessments (CT/MRI) using RECIST v.1.1, and analyzed overall and by tumor type. Dates of progression and/or death will be recorded and time-to-event analyses will be performed if sufficient data is available (overall and for individual tumor types).

11.10. Exploratory Endpoints (CTC and Exosomes)

Analysis of these parameters will be dependent upon the observed results.

11.11. PK Analysis

Following attainment of PK parameters by the non-compartmental analysis (NCA) method, the following exposure measures and pharmacokinetic parameters will be included for assessment: C_{max}, T_{max}, $t_{1/2}$, $AUC_{0-oo,}$ clearance, distribution volumes and their intra- and inter-case variability. PK parameters will be included for the main metabolite, mono-demethylated onapristone, if possible.

All PK assessments for onapristone and the main metabolite, mono-demethylated onapristone, will be presented in data listings by subject and tabulated by extended-release vs. immediate-release and by timepoint (acute vs. repeated chronic exposure).

Actual sampling time points relative to dosing will be used for the non-compartmental analyses. A unique concentration range will be used for all subjects to provide visually comparable plots. Individually-derived pharmacokinetic parameters will be listed by subject and will be summarized by means of tabulated descriptive statistics.

It is anticipated that the PK analysis will contribute to future dose selection by elucidating:

- The PK of oral extended-release dosing.
- Estimated bioavailability.
- The PK of the main metabolite.
- Quantification of M1 and M2 and presence of other metabolites.
- An indirect comparison of formulations of onapristone (immediate vs. extended release).
- PK of repeated dosing by PK population analysis and dose level simulation.

11.12. Safety Evaluation

Safety evaluations will include the following assessments at screening and throughout the study:

- AEs.
- Clinical laboratory results (including chemistry, clotting, liver function and hematology).
- *Physical examinations.*
- *Vital signs.*
- *12-lead ECG.*
- *Prior and concomitant medications.*

All safety analyses will be descriptively summarized based on the safety population.

AEs/SAEs will be coded and tabulated using the current version of Medical Dictionary for Regulatory Activities (MedDRA version 16.0).

Concomitant medications will be coded using the current WHODrug dictionary.

11.12.1. Adverse Events

AEs will be coded into standard terminology from the verbatim terms based on the MedDRA dictionary. Each AE will be classified by system organ class (SOC) and preferred term. All AEs along with the coded terms will be listed.

Treatment-emergent adverse events (TEAEs) are defined as those AEs that started on or after the first dose of study medication or that worsened after the first dose of study medication. Only TEAEs will be summarized. The incidence of TEAEs will be presented by the number and percent of patients who experienced the TEAE.

Each patient will be counted only once in the incidence for each term (overall incidence, System Organ Class [SOC] or preferred term). The incidence of treatment-related TEAEs will be summarized by SOC and preferred term. The incidence of TEAEs leading to study withdrawal or dose reduction, the incidence of serious TEAEs, and the incidence of treatment-related SAEs will be tabulated similarly.

In addition, the incidence of TEAEs will be summarized by SOC, preferred term, and maximum intensity based on CTCAE grade, by study cohort and overall, where possible.

11.12.2. Clinical Laboratory Evaluation

Clinical laboratory results including chemistry, liver function, hematology, clotting and urinalysis will be collected per the Schedules of Assessments (see Appendix 1 and Appendix 2).

Laboratory results will be tabulated as necessary or presented as per-patient listings.

11.12.3. Physical Examination

Physical examination data will be summarized descriptively by scheduled time point.

11.12.4. Vital Signs

Vital signs data will be summarized descriptively by scheduled time point.

Mean change and mean percentage change from baseline will be summarized by descriptive statistics, as appropriate.

11.12.5. 12-Lead ECG

12-lead ECG will be categorized as 'normal', 'abnormal, not clinically significant', or 'abnormal, clinically significant' and summarized descriptively.

Intervals of the cardiac cycle will be presented by descriptive statistics.

Mean change and mean percent change from baseline will be summarized by descriptive statistics, as appropriate.

Any abnormal ECG findings will be listed on a per-patient basis.

11.12.6. Physical Examination

Patients with any abnormal, clinically significant findings in the physical examination evaluation will be listed.

11.12.7. Prior and Concomitant Medications

Prior medications are defined as medications that stopped before the date of first dose of study medication. Concomitant medications are defined as any medications that started on or after the first dose of study medication.

All prior and concomitant medications will be assigned a generic name and a drug class based on the World Health Organization (WHO) Dictionary. Prior and concomitant medications will be listed and summarized by study cohort and drug class, as appropriate.

11.13. Efficacy

Tumor assessments (CT/MRI) will be assessed using RECIST v.1.1, by dose level, overall and by tumor type.

11.14. Protocol Deviations

Protocol deviations are defined as deviations from the procedures outlined in the protocol. Major protocol violations, such as significant non-compliance or other serious unforeseen violations deemed to invalidate the data collected in lieu of the purpose of the study will lead to exclusion of data from analysis. In case of minor protocol violations data will not be excluded from the data analysis.

All decisions regarding the type of deviations (major or minor) will be made prior to commencing the final analysis on the final locked database. A listing of all patients with protocol deviations will be presented.

12. STUDY MANAGEMENT

12.1. Approval and Consent

12.1.1. Regulatory Guidelines

This study will be performed in accordance with the EU Clinical Trials Directive, French Law (Public Health Code), the guidelines of the ICH, and the most recent guidelines of the Declaration of Helsinki (see Appendix 3).

12.1.2. CPP/Independent Ethics Committee

Conduct of the study must be approved by an appropriately constituted Independent Ethics Committee (IEC)/ Comite de Protection des Personnes (CPP). Approval is required for the study protocol, IB, protocol amendments, informed consent forms, patient information sheets, and any advertising materials. No study drug will be shipped to the study site until written CPP authorization has been received by the sponsor or its representative.

12.1.3. Informed Consent

For each study patient, written informed consent will be obtained prior to any protocol-related activities. As part of this procedure, the principal investigator or one of his/her associates must explain orally and in writing the nature, duration, and purpose of the study, and the action of the study drug in such a manner that the patient and (if applicable) appointed guardian are aware of the potential risks, inconveniences, or adverse effects that may occur. They should be informed that the patient may withdraw from the study at any time. They will receive all information that is required by the regulatory authorities and ICH guidelines. Biotrial will provide the sponsor with a copy of the CPP-approved informed consent form prior to the start of the study.

12.2. Protocol Amendments and Other Changes in Study Conduct

Changes to this protocol that meet the criteria of a substantial amendment in accordance with European Guidance (2010/C 82/01) require a protocol amendment that must be approved by the sponsor, the investigator(s), CPP and regulatory authorities before implementation.

The requirements for approval of the substantial changes should in no way prevent any immediate action from being taken by the investigator or by the sponsor in the interest of preserving the safety of all patients included in the study.

Amendments affecting only administrative aspects of the study that meet the criteria of a non-substantial amendment in accordance with European Guidance (2010/C 82/01) do not require formal protocol amendments or CPP approval, but the CPP must be kept informed of such administrative changes, and a log of such changes will be maintained in the case that an audit is required. The sponsor must be consulted and approve of such changes prior to their implementation.

No changes in this protocol can be made without the sponsor's written approval.

12.3. Discontinuation of the Study by the Sponsor

The sponsor reserves the right to discontinue the study for safety or administrative reasons at any time. Should the study be terminated and/or the site closed for whatever reason, all documentation and study medication pertaining to the study must be returned to the sponsor or its representative.

12.4. Study Documentation

By signing the protocol and other France-specific regulatory forms, the principal investigator acknowledges that he/she has received a copy of the Onapristone IB 2013 and assures the sponsor that he/she will comply with the protocol and French regulations concerning the conduct of clinical trials and data collection.

The investigator/Biotrial will supply the sponsor with the following documents:

- Original, France-specific regulatory forms and a signed Form FDA 1572
- Curricula vitae for all investigators listed on Form FDA 1572 and, if applicable, other forms
- Copy of principal investigator's medical license/medical registration number
- Signed protocol signature page
- List of CPP members and their occupations/affiliations or multiple assurance number
- Letter indicating CPP approval to conduct the protocol
- Copy of CPP-approved informed consent form
- Disclaimer if the investigator is a CPP member
- Laboratory certification records and reference ranges for local laboratory

The sponsor will supply the investigator with the following documents:

- Clinical study protocol
- Investigator's brochure
- Sample informed consent form
- CRFs/instruction manual
- Laboratory certification records and reference ranges for central laboratory
- Study Procedures Manual

12.5. Data Handling

No data are to be recorded directly on the CRFs (i.e. the CRF is not to be considered as source data). Data will be recorded in a FDA CFR Part 11-compliant eCRF.

Data reported on the CRF that are derived from source documents should be consistent with the source documents, or the discrepancies must be explained.

The investigator should agree to have completed and signed CRFs available for inspection by the clinical monitor at the time of each scheduled monitoring visit.

The Investigator will ensure that the confidentiality of the patients' data will be preserved. On CRFs or any other documents submitted to the Sponsor, the patients will not be identified by their names, but by an identification system. Documents not for submission to the Sponsor, *e.g.* the confidential patient identification code and the signed informed consent forms, will be maintained by the Investigator in strict confidence for as long as is requested by local regulations.

Deviations from the protocol should not occur. If deviations occur, the Investigator must inform the monitor, and the implications of the deviation must be reviewed and discussed with the sponsor. Protocol deviations are recorded in the CRF. Major deviations or deviations that include several patients must be documented in a Note to File, stating the reason and date, the action taken, and the impact on the patient and/or the study. The documentation must be kept in the Investigator's File and the Trial Master File.

12.6. Study Monitoring and Auditing

This study will be monitored by the clinical research personnel employed by the sponsor or its representative. Monitoring will include personal visits and telephone communication to assure that the investigation is conducted according to the protocol and in order to comply with guidelines of GCP. On-site review of CRFs will include a review of forms for completeness and clarity, and consistency with source documents available for each patient. Note that a variety of original documents, data, and records will be considered as source documents in this study. The CRF itself is not to be used as a source document under any circumstances.

Medical advisors and clinical research associates or assistants may request to witness patient evaluations occurring as part of this protocol. The investigator and appropriate personnel will be periodically requested to attend meetings/workshops organized by the sponsor to assure acceptable protocol execution. The study may be audited by the sponsor, its designee or by regulatory authorities. If such an audit occurs, the investigator must agree to allow access to required patient records. By signing this protocol, the investigator grants permission to personnel from the sponsor, its representatives, and appropriate regulatory authorities for on-site monitoring of all appropriate study documentation, as well as on-site review of the procedures employed in CRF generation, where clinically appropriate.

12.7. Retention of Records

The investigator agrees to conduct the study according to Good Clinical Practice (GCP) and comply with all applicable federal, state, and local laws and regulations relating to the privacy of patient health information, including, but not limited to, the EU Clinical Trials Directive and relevant sections of the French Public Health Code. The investigator shall ensure that study patients authorize the use and disclosure of protected health information in accordance with the EU Clinical Trials Directive, French and EU Law and in a form satisfactory to the sponsor.

The investigator must arrange for retention of study records at the site. The nature of the records and the duration of the retention period must meet the requirements of the relevant regulatory authority. In addition, because this is an international study, the retention period must meet the requirements of the most stringent authority. The investigator should take measures to prevent accidental or premature destruction of these documents.

12.8. Use of Study Findings

By signing the study protocol, the investigator agrees to the use of results of the study for the purposes of national and international regulatory filings and registration of onapristone. If necessary, the authorities will be notified of the investigator's name, address, qualifications, and extent of involvement. Reports covering clinical and biometric aspects of the study will be prepared by the sponsor or its representative.

12.9. Publication

The sponsor intends to publish the results of this study upon completion of the analysis and/or clinical study report (CSR). The Sponsor reserves the right to name as authors members of staff at the investigational site in the case that their contribution to the research is significant. All authors will be required to review and agree upon the content of the draft publication prior to its submission to a peer-reviewed congress, journal or posting on the Sponsor's website.

l3. REFERENCES

Allan GF, Tsai SY, Tsai MJ, and O'Malley BW. Ligand-dependent conformational changes in the progesterone receptor are necessary for events that follow DNA binding. PNAS 1992; 89:11750-11754.

Arnett-Mansfield RL, deFazio A, Wain GV, et al. Relative Expression of Progesterone Receptors A and B in Endometrioid Cancers of the Endometrium. Cancer Res 2001;61:4576-4582.

Arnett-Mansfield RL, Graham JD, Hanson AR et al. Focal Sub-nuclear Distribution of Progesterone Receptor Is Ligand Dependent and Associated with Transcriptional Activity. Molecular Endocrinology 2007; 21(1): 14-29.

Beck CA, Zhang Y, Weigel N et al. Two Types of Anti-progestins Have Distinct Effects on Site-specific Phosphorylation of Human Progesterone Receptor. The Journal of Biological Chemistry 1996; 271(2): 1209 -1217.

Benagiano G, Bastianelli C and Farris M. Selective progesterone receptor modulators 3: use in oncology, endocrinology and psychiatry. Expert Opin. Pharmacother. (2008) 9(14):2487-2496.

Cameron S, Critchley HOD, Buckley CH. et al. The effect of post-ovulatory administration of onapristone on the development of a secretory endometrium. Human reproduction: 11(1): 40-49 (1996).

Cameron S, Glasier AF, Narvekar N et al. The effects of onapristone on postmenopausal endometrium. Steroids; 68: 1053-1059 (2003)

Clemm DL, Sherman S, Boonyaratanakornkit V et al. Differential Hormone Dependent Phosphorylation of Progesterone Receptor A and B Forms Revealed by a Phosphoserine Site-Specific Monoclonal Antibody. Molecular Endocrinology 2000; 14 (1): 52-65.

Croxatto H, Salvatierra AM, Fuentealba B. Effect of the antiprogestin onapristone on follicular growth in women. Human Reproduction; 9(8): 1442-1447 (1994).

EMA. Committee for Human Medicinal Products (CHMP): Guideline on bioanalytical

Method Validation , EMEA/CHMP/EWP/192217/2009, 21 July 2011

FDA. Guidance for Industry: Bioanalytical Method Validation, U.S. Department of Health and Human Services, FDA. May 2001.

Goyeneche AA, Caron RW, Telleria CM. Mifepristone inhibits ovarian cancer cell growth in vitro and in vivo. Clin Cancer Res 2007; 13: 3370-9.

Graham JD, Hanson AR, Croft AJ et al. Nuclear matrix binding is critical for progesterone receptor movement into nuclear foci. The FASEB Journal 2009; 23: 543-556.

Investigator's Brochure for Onapristone. Arno Therapeutics, Ed. 1. 2013 Version 1.0

Jang GR and Benet LZ. Antiprogestin Pharmacodynamics, Pharmacokinetics, and Metabolism; Implications for Their Long-Term Use. Journal of Pharmacokinetics and Biopharmaceutics; 25(6): 647-672 (1997).

Jonat W,Giurescu M, Robertson JFR. The clinical efficacy of progesterone antagonist in breast cancer. Endocrine Therapy of Breast Cancer. Editors: J.F.R. Robertson, R.I. Nicholson, D.F. Hayes - Martin Dunitz, Chapter 8:117-124 (2002).

Kim J, Kurita T and Bulun SE. Progesterone Action in Endometrial Cancer, Endometriosis, Uterine Fibroids, and Breast Cancer. Endocrine Reviews 2013; 34(1):130-162

Kurita T, Young P, Brody JR et al. Stromal progesterone receptors mediate the inhibitory effects of progesterone on estrogen-induced uterine epithelial cell deoxyribonucleic acid synthesis. Endocrinology 1998; 139:11, 4708.

Lange C et al. Progesterone Receptor Action: Translating Studies in Breast Cancer Models to Clinical Insights. Chapter 7 in Innovative Endocrinology of Cancer 2010; 94-111.

Mote P and Clarke C. Relative expression of progesterone receptors A and B in premalignant and invasive breast lesions. Breast Cancer Res 2000;2(S1):21-2.

Mueller MD, Vigne JL, Pritts EA et al. Progestins activate vascular endothelial growth factor gene transcription in endometrial adenocarcinoma cells. Fertility and Sterility 2003;79 (2); 386- 392.

Pierson-Mullany LK and Lange CA. Phosphorylation of Progesterone Receptor Serine 400 Mediates Ligand-Independent Transcriptional Activity in Response to Activation of Cyclin-Dependent Protein Kinase 2. Mol. Cell. Biol. 2004; 24(24):10542.

Puri CP, Elger WG, Pongubala JMR. Menstruation by Antiprogesterone ZK98299 in Cycling Bonnet Monkeys. Contraception 35 (4) 1987: 409-421.

Puri CP, Patil RK, Elger WAG. Effects of Progesterone Antagonist ZK 98.299 on Early Pregnancy and Foetal Outcome in Bonnet Monkeys. Contraception; 41(2): 197-205 (1990)

Qiu M, Olsen A, Faivre E, et al. Mitogen-Activated Protein Kinase Regulates Nuclear Association of Human Progesterone Receptors. Molecular Endocrinology 2003 17(4):628-642.

Robertson JRR, Willsher PC, Winterbottom L et al. Onapristone, a Progesterone Receptor Antagonist, as First-line Therapy in Primary Breast Cancer. European Journal of Cancer; 35(2): 214-218 (1999).

Rocereto TF, Saul HM, Aikins JA Jr, Paulson J. Phase II study of mifepristone (RU486) in refractory ovarian cancer. Gynecol Oncol 2000 ; 77 : 429 -32.

Rudereanu D, Vering A, Stegmilller M and Bender HG. Wirkung des Progesteronantagonisten Onapristone in einem xenotransplantierten Endometriumkarzinom. In Gynakologie und Geburtshilfe 1993; Ed. Krebs D and Berg D Springer-Verlag, Ch.VIII. Onkologie- Grundlagen und Chemotherapie,1993:1065-7.

Scarpin KM, Graham D, Mote PA et al. Progesterone action in human tissues: regulation by progesterone receptor (PR) isoform expression, nuclear positioning and coregulator expression. Nuclear Receptor Signaling 2009; 7: 1-13.

Singh MM, Fahnrich M, Hasan SH et al. Reversibility of Antigestagenic Action of Antiprogestin Onapristone by Exogenus Progestagens During Early Pregnancy in Guinea Pig. Contraception; 52: 187-193 (1995).

Snyder BW, Reel JR, Winneker RC et al. Effect of Epostane, ZK98299 and ZK98734 on the Interruption of Pregnancy in the Rat. Biol Reprod 40 (1989): 549-554.

Zurth C and Kagels F. Determination of onapristone and its N-desmethyl metabolite in

human plasm or serum by HPLC. J Chromatogr 532 (1990):115-23.

14. APPENDICES

Appendix 1 : Schedule of Assessments

Appendix 2 : Schedule of Blood and Urine Sampling for Pharmacokinetics

Appendix 3 : Declaration of Helsinki

Appendix 4 : Substrates of Cytochrome P450 3A4

Appendix 5 : Hy's Law

Appendix 6 : Tissue Block Guidelines

Appendix 7 : Processing Instructions for Processing of Ascitic or Pleural Fluid to Produce a Pellet for IHC

Appendix 8 : Cockroft-Gault Equation for Calculated Creatinine Clearance

APPENDIX 1. SCHEDULE OF ASSESSMENTS

Visits	Screening	Baseline	Treatment[6]												Follow-up
Assessment	Day -21 to 0	Day 1	Day 2	Day 8	Day 15	Day 22	Day 29	Day 36	Day 43	Day 50	Day 57	Every 2 Wks	Every 4 Wks	Every 8 Wks	30 Days after last dose
Obtain informed consent	X														
Inclusion/exclusion criteria	X	X													
Demographics	X														
Medical history	X														
Physical examination[1]	X	X		X			X				X		X		X
Vital signs	X	X		X			X				X		X		X
ECOG performance status	X	X		X			X				X		X		X
12-lead ECG	X	X			X		X				X	X			X
Hematology[2]	X	X					X				X		X		X
Coagulation[2]	X	X					X				X		X		X
Blood chemistry[2]	X	X					X				X		X		X
LFTs[2,5]	X	X		X	X	X	X	X	X	X	X	X			X
Serum cortisol levels	X						X								
Serology and virology[10]	X														
Urinalysis	X	X					X				X		X		X
Randomization		X													
Onapristone dosing		X		X	X	X	X	X	X	X	X	X			
PK samples: Urine[7]		X	X												
PK samples: Blood[8]		X	X	X			X				X				
Blood for CTCs		X					X				X		X		X[9]
Blood for exosomes		X													
Tumor assessments[3]	X										X			X[3]	X[9]
Tumor blocks or biopsy	X														
Concomitant medications	X	X		X	X	X	X	X	X	X	X	X			X
Record AEs[4]	X	X		X	X	X	X	X	X	X	X	X			X

[1] Complete physical examination including height and weight will be performed at the screening visit and an abbreviated (targeted) physical examination including weight will be performed at other treatment visits and at the follow-up visit.

[2] In the event of an unexplained clinically significant abnormal laboratory test value, the test should be repeated immediately and followed up until it has returned to the normal range and/or an adequate explanation of the abnormality is found.

[3] Tumor assessments (CT/MRI) will be performed every 8 weeks after start of treatment until progression.
[4] AEs will be collected from the time of signed IC until follow-up. AEs will only be classed as treatment-emergent if they occur after first drug administration.
[5] If a Grade 3-4 LFT elevation occurs, a PK sample will be drawn as soon as possible, as well as a urine specimen for PK. This applies to the entire treatment period, even outside of the 8 week safety observation.
[6] Every effort should be made to perform the post-D1 study visits on the planned days. However, a window of +/- 1 day is acceptable during the initial 57 days of therapy and +/-2 days after day 57.
[7] PK urine samples to be collected over the following ranges: pre-dose, 0-1, 1-3, 3-6, 6-12, and 12-24 hours post-dose for Institut Curie patients only.
[8] 24H PK for 100mg immediate release onapristone patients only.
[9] Non-PD patients only.
[10] Historic serology and virology values should be documented; no new sampling and testing required.

APPENDIX 2. SCHEDULE OF BLOOD AND URINE SAMPLING FOR PHARMACOKINETICS (INCLUDING TOTAL BLOOD VOLUMES IN COMBINATION WITH SAFETY LABORATORY BLOOD DRAWS)

Assessment	Screen	Day 1	Day 2	Day 8	Day 15	Day 22	Day 29	Day 36	Day 43	Day 50	Day 57	Total
PK blood samples:												
Pre-dose		2.6 mL		2.6 mL			2.6 mL				2.6 mL	
1 hour post-dose		2.6 mL										
2 hours post-dose		2.6 mL										
4 hours post-dose		2.6 mL										
6 hours post-dose		2.6 mL										
8 hours post-dose		2.6 mL										
12 hours post-dose		2.6 mL										
24 hours post-dose[1]			2.6 mL									
PK urine samples[2]:												
Pre-dose		X										
0-1 hours post-dose		X										
1-3 hours post-dose		X										
3-6 hours post-dose		X										
6-12 hrs post-dose		X										
12-24 hrs post-dose		X										
Hematology	4 mL	4 mL					4 mL				4 mL	
Coagulation	9 mL	9 mL					9 mL				9 mL	
Chemistry[3]	15 mL	15 mL					15 mL				15 mL	
LFTs[4]	1 mL	1 mL			1 mL	1 mL	1 mL	1 mL	1 mL	1 mL	1 mL	
Cortisol	1 mL						1 mL				1 mL	
Blood for CTCs		10 mL					10 mL				10 mL	
Blood for exosomes		2.5 mL										
**Total blood volume per day:**	**30 mL**	**59.7 mL**	**2.6 mL**	**2.6 mL**	**1 mL**	**1 mL**	**42.6 mL**	**1 mL**	**1 mL**	**1 mL**	**42.6 mL**	**185.1 mL**

[1] 24 hour post-dose sample only in patients receiving 100mg immediate release.

[2] Institut Curie patients only.

[3] Serum chemistry includes electrolytes, fasting blood glucose and lipid profile.

[4] If a Grade 3-4 LFT elevation occurs, a PK sample will be drawn as soon as possible, as well as urine specimens for PK per the schedule described for day 1. This applies to the entire treatment period, even outside of the 8 week safety observation period.

APPENDIX 3. DECLARATION OF HELSINKI

WORLD MEDICAL ASSOCIATION DECLARATION OF HELSINKI

Ethical Principles for Medical Research Involving Human Patients

Adopted by the 18th WMA General Assembly, Helsinki, Finland, June 1964, and amended by the:

29th WMA General Assembly, Tokyo, Japan, October 1975

35th WMA General Assembly, Venice, Italy, October 1983

41st WMA General Assembly, Hong Kong, September 1989

48th WMA General Assembly, Somerset West, Republic of South Africa, October 1996

52nd WMA General Assembly, Edinburgh, Scotland, October 2000

53th WMA General Assembly, Washington 2002 (Note of Clarification on paragraph 29 added)

55th WMA General Assembly, Tokyo 2004 (Note of Clarification on Paragraph 30 added)

59th WMA General Assembly, Seoul, October 2008

A. INTRODUCTION

1. The World Medical Association (WMA) has developed the Declaration of Helsinki as a statement of ethical principles for medical research involving human patients, including research on identifiable human material and data. The Declaration is intended to be read as a whole and each of its constituent paragraphs should not be applied without consideration of all other relevant paragraphs.

2. Although the Declaration is addressed primarily to physicians, the WMA encourages other participants in medical research involving human patients to adopt these principles.

3. It is the duty of the physician to promote and safeguard the health of patients, including those who are involved in medical research. The physician's knowledge and conscience are dedicated to the fulfillment of this duty.

4. The Declaration of Geneva of the WMA binds the physician with the words, "The health of my patient will be my first consideration," and the International Code of Medical Ethics declares that, "A physician shall act in the patient's best interest when providing medical care."

5. Medical progress is based on research that ultimately must include studies involving human patients. Populations that are underrepresented in medical research should be provided appropriate access to participation in research.

6. In medical research involving human patients, the well-being of the individual research patient must take precedence over all other interests.

7. The primary purpose of medical research involving human patients is to understand the causes, development and effects of diseases and improve preventive, diagnostic and therapeutic interventions (methods, procedures and treatments). Even the best current interventions must be evaluated continually through research for their safety, effectiveness, efficiency, accessibility and quality.

8. In medical practice and in medical research, most interventions involve risks and burdens.

9. Medical research is patient to ethical standards that promote respect for all human patients and protect their health and rights. Some research populations are particularly vulnerable and need special protection. These include those who cannot give or refuse consent for themselves and those who may be vulnerable to coercion or undue influence.

10. Physicians should consider the ethical, legal and regulatory norms and standards for research involving human patients in their own countries as well as applicable international norms and standards. No national or international ethical, legal or regulatory requirement should reduce or eliminate any of the protections for research patients set forth in this Declaration.

B. PRINCIPLES FOR ALL MEDICAL RESEARCH

11. It is the duty of physicians who participate in medical research to protect the life, health, dignity, integrity, right to self-determination, privacy, and confidentiality of personal information of research patients.

12. Medical research involving human patients must conform to generally accepted scientific principles, be based on a thorough knowledge of the scientific literature, other relevant sources of information, and adequate laboratory and, as appropriate, animal experimentation. The welfare of animals used for research must be respected.

13. Appropriate caution must be exercised in the conduct of medical research that may harm the environment.

14. The design and performance of each research study involving human patients must be clearly described in a research protocol. The protocol should contain a statement of the ethical considerations involved and should indicate how the principles in this Declaration have been addressed. The protocol should include information regarding funding, sponsors, institutional affiliations, other potential conflicts of interest, incentives for patients and provisions for treating and/or compensating patients who are harmed as a consequence of participation in the research study. The protocol should describe arrangements for post-study access by study patients to interventions identified as beneficial in the study or access to other appropriate care or benefits.

15. The research protocol must be submitted for consideration, comment, guidance and approval to a research ethics committee before the study begins. This committee must be independent of the researcher, the sponsor and any other undue influence. It must take into consideration the laws and regulations of the country or countries in which the research is to be performed as well as applicable international norms and standards but these must not be allowed to reduce or eliminate any of the protections for research patients set forth in this Declaration. The committee must have the right to monitor ongoing studies. The researcher must provide monitoring information to the committee, especially information about any SAEs. No change to the protocol may be made without consideration and approval by the committee.

16. Medical research involving human patients must be conducted only by individuals with the appropriate scientific training and qualifications. Research on patients or healthy volunteers requires the supervision of a competent and appropriately qualified physician or other health care professional. The responsibility for the protection of research patients must always rest with the

physician or other health care professional and never the research patients, even though they have given consent.

17. Medical research involving a disadvantaged or vulnerable population or community is only justified if the research is responsive to the health needs and priorities of this population or community and if there is a reasonable likelihood that this population or community stands to benefit from the results of the research.

18. Every medical research study involving human patients must be preceded by careful assessment of predictable risks and burdens to the individuals and communities involved in the research in comparison with foreseeable benefits to them and to other individuals or communities affected by the condition under investigation.

19. Every clinical trial must be registered in a publicly accessible database before recruitment of the first patient.

20. Physicians may not participate in a research study involving human patients unless they are confident that the risks involved have been adequately assessed and can be satisfactorily managed. Physicians must immediately stop a study when the risks are found to outweigh the potential benefits or when there is conclusive proof of positive and beneficial results.

21. Medical research involving human patients may only be conducted if the importance of the objective outweighs the inherent risks and burdens to the research patients.

22. Participation by competent individuals as patients in medical research must be voluntary. Although it may be appropriate to consult family members or community leaders, no competent individual may be enrolled in a research study unless he or she freely agrees.

23. Every precaution must be taken to protect the privacy of research patients and the confidentiality of their personal information and to minimize the impact of the study on their physical, mental and social integrity.

24. In medical research involving competent human patients, each potential patient must be adequately informed of the aims, methods, sources of funding, any possible conflicts of interest, institutional affiliations of the researcher, the anticipated benefits and potential risks of the study and the discomfort it may entail, and any other relevant aspects of the study. The potential patient must be informed of the right to refuse to participate in the study or to withdraw consent to participate at any time without reprisal. Special attention should be given to the specific information needs of individual potential patients as well as to the methods used to deliver the information. After ensuring that the potential patient has understood the information, the physician or another appropriately qualified individual must then seek the potential patient's freely-given informed consent, preferably in writing. If the consent cannot be expressed in writing, the non-written consent must be formally documented and witnessed.

25. For medical research using identifiable human material or data, physicians must normally seek consent for the collection, analysis, storage and/or reuse. There may be situations where consent would be impossible or impractical to obtain for such research or would pose a threat to the validity of the research. In such situations the research may be done only after consideration and approval of a research ethics committee.

26. When seeking informed consent for participation in a research study the physician should be particularly cautious if the potential patient is in a dependent relationship with the physician or may consent under duress. In such situations the informed consent should be sought by an appropriately qualified individual who is completely independent of this relationship.

27. For a potential research patient who is incompetent, the physician must seek informed consent from the legally authorized representative. These individuals must not be included in a research study that has no likelihood of benefit for them unless it is intended to promote the health of the population represented by the potential patient, the research cannot instead be performed with competent persons, and the research entails only minimal risk and minimal burden.

28. When a potential research patient who is deemed incompetent is able to give assent to decisions about participation in research, the physician must seek that assent in addition to the consent of the legally authorized representative. The potential patient's dissent should be respected.

29. Research involving patients who are physically or mentally incapable of giving consent, for example, unconscious patients, may be done only if the physical or mental condition that prevents giving informed consent is a necessary characteristic of the research population. In such circumstances the physician should seek informed consent from the legally authorized representative. If no such representative is available and if the research cannot be delayed, the study may proceed without informed consent provided that the specific reasons for involving patients with a condition that renders them unable to give informed consent have been stated in the research protocol and the study has been approved by a research ethics committee. Consent to remain in the research should be obtained as soon as possible from the patient or a legally authorized representative.

30. Authors, editors and publishers all have ethical obligations with regard to the publication of the results of research. Authors have a duty to make publicly available the results of their research on human patients and are accountable for the completeness and accuracy of their reports. They should adhere to accepted guidelines for ethical reporting. Negative and inconclusive as well as positive results should be published or otherwise made publicly available. Sources of funding, institutional affiliations and conflicts of interest should be declared in the publication. Reports of research not in accordance with the principles of this Declaration should not be accepted for publication.

C. ADDITIONAL PRINCIPLES FOR MEDICAL RESEARCH COMBINED WITH MEDICAL CARE

31. The physician may combine medical research with medical care only to the extent that the research is justified by its potential preventive, diagnostic or therapeutic value and if the physician has good reason to believe that participation in the research study will not adversely affect the health of the patients who serve as research patients.

32. The benefits, risks, burdens and effectiveness of a new intervention must be tested against those of the best current proven intervention, except in the following circumstances:

- The use of placebo, or no treatment, is acceptable in studies where no current proven intervention exists; or
- Where for compelling and scientifically sound methodological reasons the use of placebo is necessary to determine the efficacy or safety of an intervention and the patients who receive placebo or no treatment will not be patient to any risk of serious or irreversible harm. Extreme care must be taken to avoid abuse of this option.

33. At the conclusion of the study, patients entered into the study are entitled to be informed about the outcome of the study and to share any benefits that result from it, for example, access to interventions identified as beneficial in the study or to other appropriate care or benefits.

34. The physician must fully inform the patient which aspects of the care are related to the research. The refusal of a patient to participate in a study or the patient's decision to withdraw from the study must never interfere with the patient-physician relationship.

35. In the treatment of a patient, where proven interventions do not exist or have been ineffective, the physician, after seeking expert advice, with informed consent from the patient or a legally authorized representative, may use an unproven intervention if in the physician's judgment it offers hope of saving life, re-establishing health or alleviating suffering. Where possible, this intervention should be made the object of research, designed to evaluate its safety and efficacy. In all cases, new information should be recorded and, where appropriate, made publicly available.

APPENDIX 4. SUBSTRATES OF CYP450 3A4

CYP3A4 inducers	CYP3A4 inhibitors	CYP3A4 substrates
rifampicin, rifabutin,<br><br>carbamazepine,phenobarbital, amobarbital,<br><br>primidone,phenytoin,<br><br>felbamate,<br><br>dexamethasone,<br><br>omeprazole,<br><br>nevirapine, efavirenz<br><br>St John's wort.	grapefruit juice,<br><br>ketoconazole, miconazole, itraconazole, fluconazole posaconazole, voriconazoIe,<br><br>erythromycin, clarithromycin, telithromycin,<br><br>verapamil, diltiazem<br><br>cyclosporine,<br><br>indinavir, saquinavir, ritonavir, nelfmavir, lopinavir, atazanavir, amprenavlr, fosamprenavir, darunavir, indinavir, delavirdine.	statins: lovastatin, atorvastatin, simvastatin<br><br>chemotherapeutic agents<br><br>azole antifungals<br><br>macrolides<br><br>SSRIs<br><br>antidepressants<br><br>antipsychotics<br><br>opioids<br><br>benzodiazepines<br><br>calcium channel blockers

NB A more extensive list is available in the onapristone IB [Onapristone IB 2013]

APPENDIX 5. HY'S LAW

Hy's law is a prognostic indicator that a pure drug-induced liver injury (DILI) leading to jaundice, without a hepatic transplant, has a case fatality rate of 10% to 50%. The law is based on observations by Hy Zimmerman, an authority on drug-induced liver injury.

Hy's Law cases have the following three components

The drug causes hepatocellular injury, generally shown by more frequent 3-fold or greater elevations above the upper limits of normal (ULN) of ALT or AST than the (nonhepatotoxic) control agent or placebo.

Among subjects showing such aminotransferase (AT) elevations, often with ATs much greater than 3xULN, some subjects also show elevation of serum total bilirubin (TBL) to >2xULN, without initial findings of cholestasis (serum alkaline phosphatase (ALP) activity >2xULN).

No other reason can be found to explain the combination of increased AT and serum TBL, such as viral hepatitis A, B, or C, preexisting or acute liver disease, or another drug capable of causing the observed injury.

As defined by The U.S. Department of Health and Human Services, Food and Drug Administration, Center for Drug Evaluation and Research (CDER) Center for Biologics Evaluation and Research (CBER) in their final document of 2009 Guidance for Industry Drug-Induced Liver Injury: Premarketing Clinical Evaluation.

References

Guidance for Industry Drug-Induced Liver Injury: Premarketing Clinical Evaluation, Final, July 2009

Downloaded from Wikipedia: http://en.wikipedia.org/wiki/Hy%27s_law

APPENDIX 6. TISSUE BLOCK GUIDELINES

For enrolment into the study, it is expected that for each patient a representative tumor sample will be available, which give a reasonable chance of reflecting the initial and current PR and APR status of the tumor. At this point in time it is unknown what impact endocrine manipulation, HER2-targeted therapy or standard chemotherapies have on the PR status of the tumor relative to the original malignancy. Current tumor specimens (relapse, metastasis biopsy) for analysis may be required in addition to the initial tumor specimen (biopsy or surgical specimen). Therefore BOTH archival tissue blocks (if available) and recently obtained tumor tissue (current) should be provided per the definitions below.

All tumor blocks/samples should be formalin-fixed, paraffin-embedded (FFPE) specimens, using standard preparation techniques. Acetic formalin alcohol (AFA) prepared specimens are acceptable for archival tissue but not for new biopsies. The following examples are provided as guidance to the investigator.

If you cannot send paraffin blocks for any reason, please send 10 unstained slides from initial and current blocks.

If a specific patient's case is unclear, please call the Medical Monitor.

**Initial Tumor (if available)**		**Current Specimen (if needed due to interim therapy or time)**	
Acceptable	Unacceptable	Acceptable	Unacceptable
• Tissue paraffin block (preferred) or 10 serial, freshly cut, unstained slides plus associated pathology report<br>• Primary tumor from time of initial diagnosis<br>• In addition, paraffin blocks corresponding to any interim sample, e.g. relapse, 1st metastasis prior to subsequent systemic therapy; synchronous metastasis	• Fine needle aspiration<br>• Cytology sample	• Tissue paraffin block (preferred) or 10 serial, freshly cut, unstained slides plus associated pathology report<br>• Sample :S6 months prior to day 1 with no interim systemic treatment (if >6 months please call medical monitor)<br>• Ascites or pleural fluid with adequate malignant cells to produce a pellet to perform IHC (See Appendix 7 for processing instructions)	• Fine needle aspiration.<br>• Cytology sample<br>• Sample taken before progression of disease from previous systemic agent<br>• Sample followed by any subsequent systemic treatment, e.g. endocrine-, chemotherapy, antibodies or kinase inhibitors

APPENDIX 7. INSTRUCTIONS FOR PROCESSING ASCITIC OR PLEURAL FLUID TO PRODUCE A PELLET FOR IHC

- After aspiration, up to 30 mL of fluid should be placed into heparinized containers and kept at 4°C.
- The specimens should not be kept under these conditions for more than 24 hours.
- If the obtained volume seems small (< 1 mL) but of good quality, the available liquid should nevertheless be sent for processing.
- Label container with study ID, site and patient ID.
- Contact Biodoxis, who will send a courier, which will collect the sample the same day.

Biodoxis contact details:
Laura Caplier, DMV
Email : Laura.caplier@biodoxis.com

BiodOxis
Parc Biocitech
102 avenue Gaston Roussel
93 230 Romainville
FRANCE
Tel: +33 (0) 1 48 43 70 77
Fax: +33 (0) 1 48 43 70 58

- Biodoxis will produce pellets, process the IHC and send the slides to the central pathologist.

APPENDIX 8. COCKROFT-GAULT EQUATION FOR CALCULATED CREATININE CLEARANCE

$$eC_{Cr} = \frac{(\text{140 - Age}) \times \text{Mass (in kilograms)} \times [0.85\ if\ Female]}{72 \times \text{Serum Creatinine (in mg/dL)}}$$

Or

$$eC_{Cr} = \frac{(\text{140 - Age}) \times \text{Mass (in kilograms)} \times Constant}{\text{Serum Creatinine (in } \mu\text{mol/L)}}$$

Where constant is 1.23 for men and 1.04 for women.
